# Kujiol A Inhibits Interferon-γ and Interleukin-2 Expression and the NFATc2 Interaction with Their Promoters in T Cells

**DOI:** 10.3390/molecules31101613

**Published:** 2026-05-11

**Authors:** Tanpitcha Yodweerapong, Rikako Yamaguchi, Ayaka Nakao, Sakihito Kitajima, Tomoo Shiba, Ken-ichi Kimura, Takao Kataoka

**Affiliations:** 1 Department of Applied Biology, Kyoto Institute of Technology, Matsugasaki, Sakyo-ku, Kyoto 606-8585, Japan; 2 The United Graduate School of Agricultural Sciences, Iwate University, 3-18-8 Ueda, Morioka 020-8550, Japan; 3 Center for Social and Biomedical Engineering, Kyoto Institute of Technology, Matsugasaki, Sakyo-ku, Kyoto 606-8585, Japan

**Keywords:** kujiol A, kujigamberol, kujigamberoic acid A, spirolactone norditerpenoid, interferon-γ, interleukin-2, cytokine, NFAT, T cell

## Abstract

Kujiol A is one of the kujigamberol-related compounds isolated from Kuji amber. We previously demonstrated that kujiol A exhibited multiple biological activities, including the inhibition of Ca^2+^ signal transduction. In the present study, we found that kujiol A prevented the transcription of interferon-γ (IFN-γ), interleukin (IL)-2, IL-4, and Fas ligand in T-box transcription factor Eomesodermin (Eomes)-transfected murine T cell lymphoma BW5147 cells stimulated with phorbol ester and ionomycin (a calcium ionophore). In the murine cytotoxic T cell line CTLL-2, kujiol A reduced ionomycin-induced increases in IFN-γ mRNA expression. Luciferase reporter assays revealed that kujiol A inhibited the transcriptional activities of nuclear factor of activated T cells (NFAT) and, to a lesser extent, nuclear factor κB in human embryonic kidney 293T cells. Kujiol A did not affect NFATc2 (also known as NFAT1) protein expression. However, a chromatin immunoprecipitation assay showed that kujiol A prevented the NFATc2 protein from interacting with the IFN-γ and IL-2 promoters in Eomes-transfected BW5147 cells. Collectively, these results demonstrate that kujiol A is a potent immunosuppressant that inhibits T cell cytokine expression by targeting the NFAT pathway.

## 1. Introduction

The pro-inflammatory cytokine interferon-γ (IFN-γ) is primarily produced by CD4^+^ T cells, CD8^+^ T cells, natural killer T cells, and other immune cells and plays an essential role in regulating immune responses [1,2,3,4]. IFN-γ is a pleiotropic cytokine that contributes to host defenses by enhancing macrophage activation, promoting antigen presentation, and directing immune responses toward a cell-mediated phenotype [1,2,3,4]. Within CD4^+^ helper T cell subsets, IFN-γ is secreted by T helper type 1 (Th1) cells, which play critical roles in cellular immunity, whereas interleukin (IL)-4, IL-5, and IL-13 are secreted by T helper type 2 (Th2) cells, which are associated with humoral and parasitic immunity [5,6,7,8]. IL-2 is a growth factor for T cells and is produced by activated T cells [9,10], whereas Th1 cells are capable of secreting IL-2 [6,8]. IL-2 promotes the proliferation, survival, and clonal expansion of T cells [11,12,13,14]. IL-2 is also involved in the development of regulatory T cells, as well as the differentiation of helper T cells and effector and memory CD8^+^ T cells [11,12,13,14].

The stimulation of T cell receptors induces the activation of multiple signaling cascades, which, in turn, activate transcription factors, including activated protein-1 (AP-1), nuclear factor κB (NF-κB), and nuclear factor of activated T cells (NFAT) [15,16,17]. In the Ca^2+^–calcineurin–NFAT pathway, increased intracellular Ca^2+^ promotes the activation of the protein phosphatase calcineurin, which dephosphorylates NFAT proteins in the cytoplasm [18,19,20]. This dephosphorylation enables NFAT proteins to translocate into the nucleus, where they bind to the promoters of various cytokines, such as IFN-γ, IL-2, and IL-4 [21,22,23,24].

Kujigamberol is an ingredient isolated from Kuji amber [25]. It has been reported to exert an anti-cancer effect on human leukemia HL-60 cells [25], an anti-allergy effect on rat basophilic leukemia RBL-2H3 cells and a guinea pig rhinitis model [26], and an anti-inflammatory effect on human umbilical vein endothelial cells (HUVECs) [27]. We recently showed that kujigamberol possesses an immunosuppressive property that prevents NFATc2 from binding to the proximal promoter regions of the IFN-γ and IL-2 genes [28].

In addition to kujigamberol, novel related compounds with unique structures have been isolated from Kuji amber. Of these, compounds with the representative skeletons of labdane, podocarpan, and spirolactone have been investigated for their biological activities [29,30,31]. While kujigamberol possesses a labdane structure, kujiol A has a podocarpan skeleton. A previous study demonstrated that kujiol A inhibited the transduction of Ca^2+^ signals in mutant yeast that were hypersensitive to Ca^2+^ signaling [29]. Furthermore, spirolactone norditerpenoid was found to exert inhibitory effects on Ca^2+^ signal transduction as well as degranulation in RBL-2H3 cells [30]. Kujigamberoic acid A has a labdane structure and was shown to inhibit the degranulation of RBL-2H3 cells [31].

Although the biological activities of kujigamberol-related compounds have been investigated, their specific target molecules in mammalian cells remain unclear. We herein compared the immunosuppressive effects of kujigamberol-related compounds on T cell cytokine expression. Among the kujigamberol-related compounds tested in the present study, the inhibitory effects of kujiol A were similar to those of kujigamberol. Therefore, we attempted to elucidate the mechanisms by which kujiol A inhibits T cell cytokine expression.

## 2. Results

### 2.1. Kujiol A Exerted Strong Inhibitory Effects on Cytokine Expression in Eomesodermin (Eomes)-Transfected BW5147 Cells

**Figure 1:**
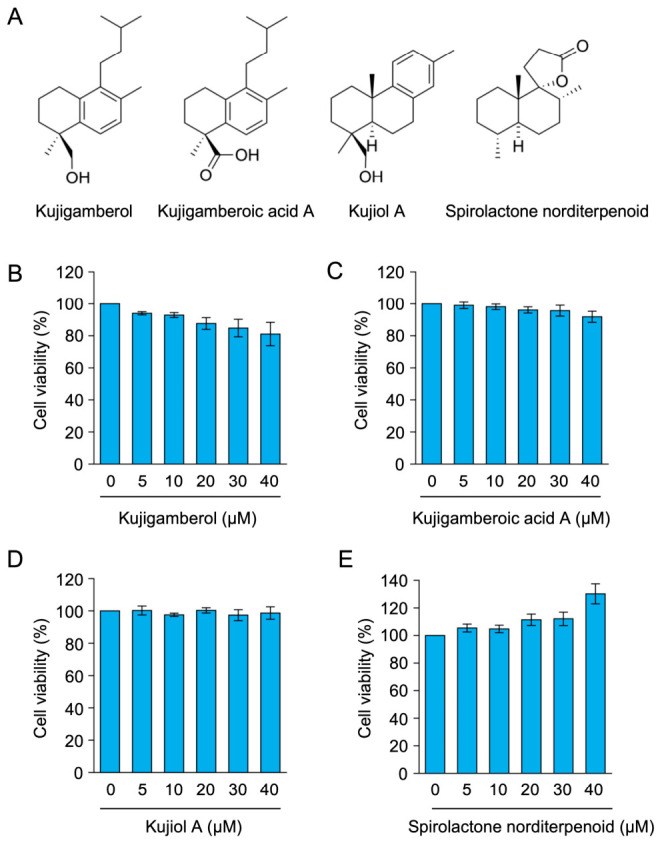
Structures of kujigamberol-related compounds and their effects on BW5147 cell viability: (**A**) Structures of kujigamberol-related compounds. (**B**–**E**) Eomes-BW5147 cells were exposed to serially diluted concentrations of kujigamberol (**B**), kujigamberoic acid A (**C**), kujiol A (**D**), or spirolactone norditerpenoid (**E**) (5, 10, 20, 30, and 40 µM) for 6 h. Cell viability (%) is represented by the mean ± standard error of the mean (SEM) (*n* = 3). No significant differences were observed from the control.

BW5147 cells are murine T cell lymphoma cells that express IL-2 but not IFN-γ [32], while BW5147 cells stably transfected with Eomes (Eomes-BW5147 cells) express IL-2, IFN-γ, IL-4, and Fas ligand mRNAs [28]. In the present study, Eomes-BW5147 cells were used as a T cell model because they express several Th1 and Th2 cytokines [28]. We selected four compounds from the methanol extract of Kuji amber (MEKA) that are present in high quantities and have structurally distinct skeletons: labdane, podocarpane, and spirolactone. Kujigamberol and kujigamberoic acid A have labdane structures, which possess a hydroxy group and a carboxy group, respectively (Figure 1A). Kujiol A has a podocarpane structure, whereas spirolactone norditerpenoid has a spirolactone structure (Figure 1A). We initially investigated non-toxic doses of kujigamberol-related compounds in Eomes-BW5147 cells. We previously showed that kujigamberol did not reduce the viability of Eomes-BW5147 cells [28]. Similar to kujigamberol (Figure 1B), three other compounds at concentrations up to 40 µM did not reduce cell viability during a 6 h incubation (Figure 1C–E).

**Figure 2:**
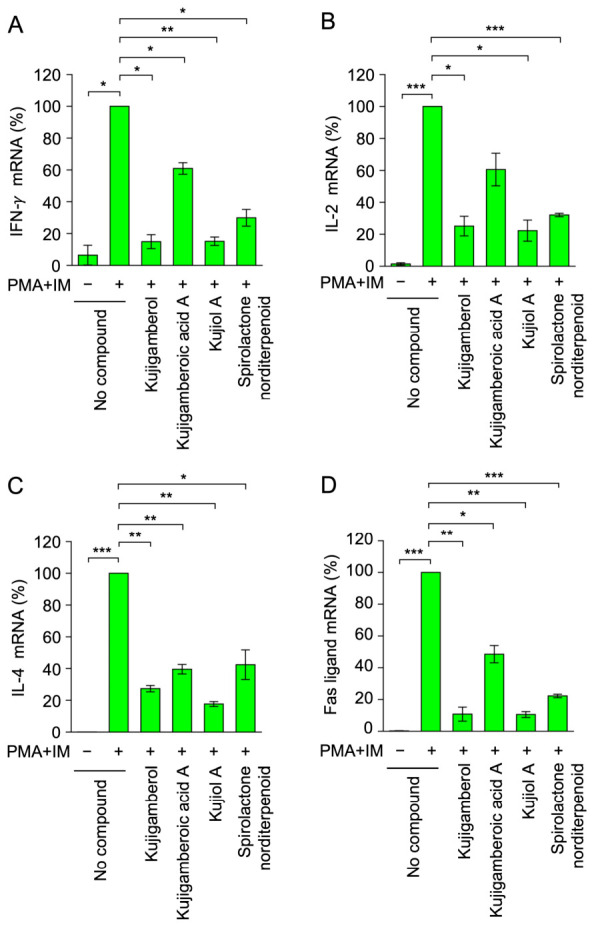
Effects of kujigamberol-related compounds on cytokine mRNA expression by BW5147 cells: (**A**–**D**) Eomes-BW5147 cells were preincubated without (no compound) or with kujigamberol-related compounds (20 µM each) for 1 h, followed by a 6 h incubation without (−) or with (+) PMA + IM (100 nM and 1 µM) in the absence or presence of compounds. IFN-γ mRNA (%) (**A**), IL-2 mRNA (%) (**B**), IL-4 mRNA (%) (**C**), and Fas ligand mRNA (%) (**D**) are represented by the mean ± SEM (*n* = 3). * *p* < 0.05, ** *p* < 0.01, and *** *p* < 0.001 indicate significant differences from PMA + IM (+).

To investigate structure-activity relationships, Eomes-BW5147 cells were exposed to each compound and then stimulated with phorbol 12-myristate 13-acetate (PMA) and ionomycin (IM). Thereafter, mRNA expression levels were measured using reverse transcription quantitative polymerase chain reaction (RT-qPCR). We previously showed that kujigamberol decreased the mRNA expression of IFN-γ, IL-2, IL-4, and Fas ligand [28]. The inhibitory activities of kujigamberol-related compounds were compared using a concentration of 20 µM because kujigamberol exerted suboptimal inhibitory effects on IFN-γ and IL-2 expression at this concentration [28]. As previously reported [28], kujigamberol inhibited the expression of IFN-γ, IL-2, IL-4, and Fas ligand mRNAs (Figure 2A–D). Furthermore, kujiol A markedly attenuated increases in the mRNA expression of IFN-γ, IL-2, IL-4, and Fas ligand (Figure 2A–D), with similar efficacy to kujigamberol. In contrast, the effects of kujigamberoic acid A and spirolactone norditerpenoid were weaker than those of kujigamberol or kujiol A (Figure 2A–D). Based on these results, we examined the biological activity and inhibitory mechanism of kujiol A.

### 2.2. Kujiol A Inhibited IFN-γ and IL-2 mRNA Levels in Eomes-BW5147 Cells

**Figure 3:**
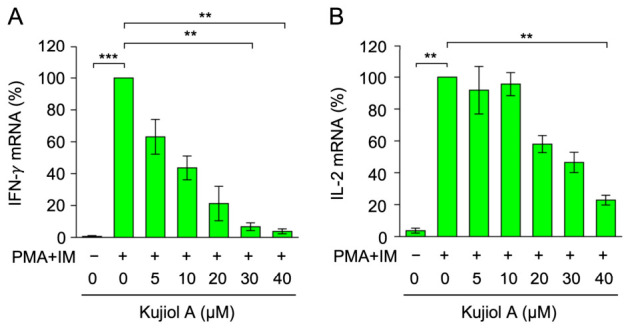
Kujiol A decreased IFN-γ and IL-2 mRNA levels in BW5147 cells: (**A,B**) Eomes-BW5147 cells were preincubated with serially diluted concentrations of kujiol A (5, 10, 20, 30, and 40 µM) for 1 h, followed by a 6 h incubation without (−) or with (+) PMA + IM (100 nM and 1 µM) in the absence or presence of kujiol A. IFN-γ mRNA (%) (**A**) and IL-2 mRNA (%) (**B**) are represented by means ± SEM (*n* = 3). ** *p* < 0.01 and *** *p* < 0.001 indicate significant differences from PMA + IM (+).

To investigate the inhibitory concentrations of kujiol A, Eomes-BW5147 cells were pretreated with kujiol A (0 to 40 µM) and stimulated with PMA and IM, and mRNA levels were then measured using RT-qPCR. Kujiol A reduced IFN-γ mRNA expression in a dose-dependent manner (Figure 3A). It also dose-dependently decreased IL-2 mRNA expression (Figure 3B). The 50% inhibitory concentration (IC_50_) values of kujiol A on IFN-γ and IL-2 mRNA expression were 7.7 and 27.2 µM, respectively, in a simple regression analysis of concentrations ranging from 5 to 40 µM (Figure 3A,B). Collectively, these results suggest that IFN-γ mRNA expression was more sensitive to kujiol A than IL-2 mRNA expression.

### 2.3. Kujiol A Preferentially Inhibited NFAT-Dependent Promoter Activity in 293T Cells

**Figure 4:**
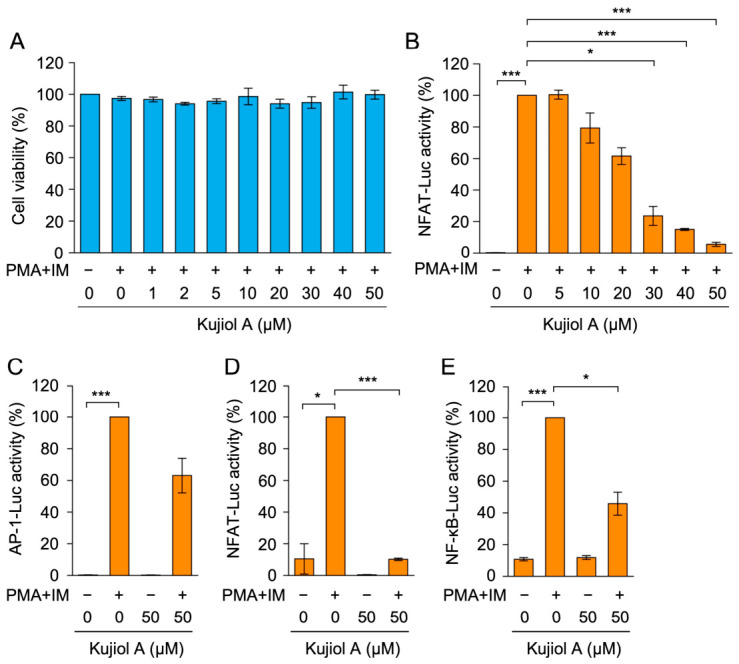
Kujiol A preferentially decreased NFAT promoter activity in 293T cells: (**A**) 293T cells were treated with serially diluted concentrations of kujiol A (1, 2, 5, 10, 20, 30, 40, and 50 µM) for 1 h, followed by a 6 h incubation without (−) or with (+) PMA + IM (100 nM and 1 µM) in the absence or presence of kujiol A. Cell viability (%) is represented by the mean ± SEM (*n* = 3). (**B**–**E**) 293T cells transiently expressing luciferase reporter vectors encoding NFAT-Luc were preincubated with serially diluted concentrations of kujiol A (5, 10, 20, 30, 40, and 50 µM) for 1 h, followed by a 6 h incubation without (−) or with (+) PMA + IM (100 nM and 1 µM) in the absence or presence of kujiol A (**B**). 293T cells transiently expressing luciferase reporter vectors encoding AP-1-Luc, NFAT-Luc, and NF-κB-Luc were preincubated without (−) or with (+) kujiol A (50 µM) for 1 h, followed by a 6 h incubation in the absence (−) or presence (+) of PMA + IM (100 nM and 1 µM) (**C**–**E**). AP-1-Luc activity (%) (**C**), NFAT-Luc activity (%) (**D**), and NF-κB-Luc activity (%) (**E**) are represented by the mean ± SEM (*n* = 3). * *p* < 0.05 and *** *p* < 0.001 indicate significant differences from PMA + IM (+).

Human embryonic kidney 293T cells were used to examine the effects of kujiol A on transcription factors that are essential for T cell cytokine expression because they have been shown to exhibit high transfection efficiency and respond to PMA and IM by increasing the expression of AP-1-Luc, NFAT-Luc, and NF-κB-Luc reporters [28]. We initially examined the effects of kujiol A on 293T cell viability. Kujiol A at concentrations up to 50 µM did not affect cell viability (Figure 4A). We then transfected 293T cells with luciferase reporter vectors, treated them with kujiol A, and stimulated them with PMA and IM. This stimulation markedly increased the luciferase reporter activity of NFAT-Luc (Figure 4B). Kujiol A diminished the increase in NFAT-Luc activity in a dose-dependent manner with an IC_50_ value of 24.7 µM (Figure 4B). In contrast, Kujiol A at 50 µM did not markedly affect AP-1-Luc activity (Figure 4C). In contrast to the strong inhibition of NFAT-Luc activity (Figure 4D), NF-κB-Luc activity was moderately suppressed by kujiol A at 50 µM (Figure 4E). These results indicate that kujiol A preferentially inhibited NFAT-dependent transcriptional activity.

### 2.4. Kujiol A Reduced IFN-γ mRNA Expression in CTLL-2 Cells

**Figure 5:**
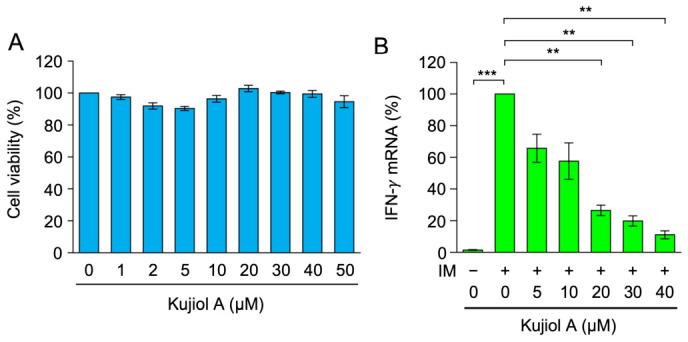
Kujiol A decreased IFN-γ mRNA levels in CTLL-2 cells: (**A**) CTLL-2 cells were exposed to serially diluted concentrations of kujiol A (1, 2, 5, 10, 20, 30, 40, and 50 µM) for 6 h. Cell viability (%) is represented by the mean ± SEM (*n* = 3). No significant differences were observed from the control. (**B**) CTLL-2 cells were preincubated with serially diluted concentrations of kujiol A (5, 10, 20, 30, and 40 µM) for 1 h, followed by a 6 h incubation without (−) or with (+) IM (1 µM) in the presence of kujiol A. IFN-γ mRNA (%) is represented by the mean ± SEM (*n* = 3). ** *p* < 0.01 and *** *p* < 0.001 indicate significant differences from IM (+).

The murine cytotoxic T cell line CTLL-2 was used to examine the effects of kujiol A in another type of T cell because it expresses IFN-γ [33,34]. CTLL-2 cells were exposed to kujiol A (0 to 50 µM) for 6 h, and cell viability was then measured. Kujiol A at concentrations up to 50 µM did not affect CTLL-2 cell viability (Figure 5A). We previously demonstrated that IFN-γ mRNA expression was lower in cells stimulated with PMA and IM than with IM alone [28]. Therefore, CTLL-2 cells were exposed to kujiol A for 1 h, followed by a 6 h stimulation with IM alone. Kujiol A decreased IFN-γ mRNA expression in CTLL-2 cells in a dose-dependent manner and at the IC_50_ value of 12.4 µM (Figure 5B). These results indicate that kujiol A inhibited IFN-γ mRNA expression in CTLL-2 cells.

### 2.5. Kujiol A Did Not Affect Nuclear NFATc2 Protein Expression in CTLL-2 Cells

**Figure 6:**
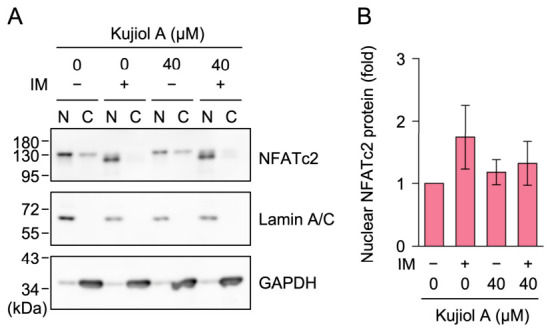
Kujiol A did not affect NFATc2 protein levels in CTLL-2 cells: (**A**,**B**) CTLL-2 cells were preincubated without (−) or with (+) kujiol A (40 µM) for 1 h, followed by a 6 h incubation without (−) or with (+) IM (1 µM) in the absence or presence of kujiol A. Representative blots of the nuclear fraction (N) and cytoplasmic fraction (C) are shown (**A**). The amount of the NFATc2 protein in the nuclear fraction was normalized by that of the lamin A/C protein. The nuclear NFATc2 protein (fold) (**B**) is represented by the mean ± SEM (*n* = 3). No significant differences were observed between 0 and 40 µM of kujiol A. Original blots are presented in Figures S1–S4.

NFATc2 is one of the five NFAT isoforms [35,36,37]. It plays a major role in IFN-γ transcription in T cells [38,39,40]. We examined the subcellular localization of the NFATc2 protein in CTLL-2 cells, which were subjected to a 1 h pretreatment with kujiol A and a 6 h treatment with IM. Nuclear and cytoplasmic fractions were prepared and analyzed by Western blotting using lamin A/C and glyceraldehyde-3-phosphate dehydrogenase (GAPDH) as markers for the nucleus and cytoplasm, respectively. In unstimulated CTLL-2 cells, a large amount of the NFATc2 protein was already detected in the nuclear fraction, whereas only a small amount was present in the cytoplasmic fraction (Figure 6A). Kujiol A did not markedly affect the nuclear amount of the NFATc2 protein in the absence or presence of IM stimulation (Figure 6A,B). Kujiol A itself did not appear to affect the electrophoretic mobility of the NFATc2 protein (Figure 6A). However, IM stimulation slightly increased the electrophoretic mobility of the NFATc2 protein (Figure 6A), suggesting that IM stimulation induced the dephosphorylation of the NFATc2 protein.

### 2.6. Kujiol A Did Not Affect NFATc2 Protein Levels in Eomes-BW5147 Cells

**Figure 7:**
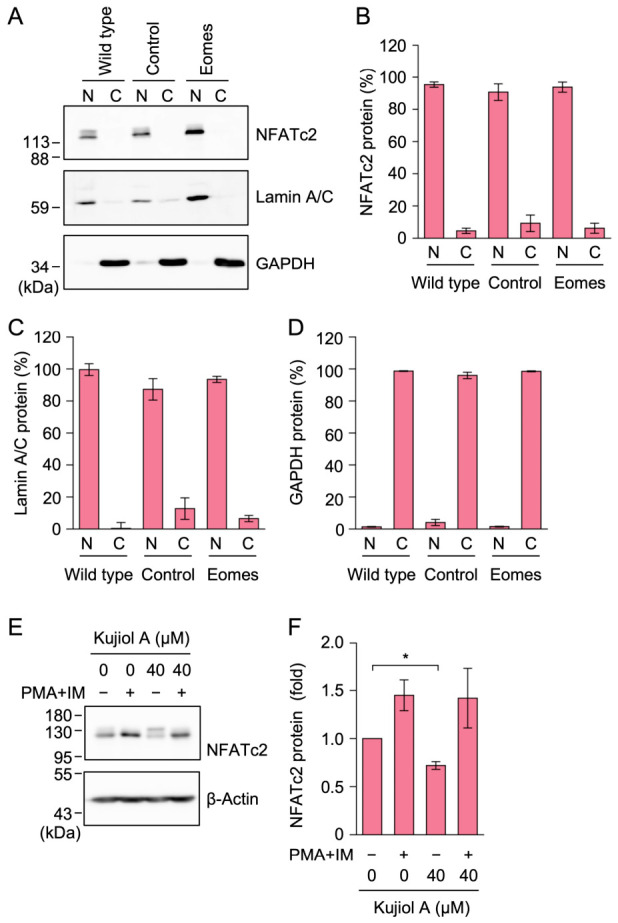
Kujiol A did not markedly affect NFATc2 protein levels in BW5147 cells: (**A**–**D**) The nuclear fraction (N) and cytoplasmic fraction (C) were prepared from BW5147 cells (wild type), empty vector-transfected BW5147 cells (control), and Eomes-BW5147 cells (Eomes) and were then subjected to Western blotting. Panel (**A**) shows representative blots from four independent experiments. The nuclear and cytoplasmic levels of the NFATc2 protein (%) (**B**), lamin A/C protein (%) (**C**), and GAPDH protein (%) (**D**) are represented by the mean ± SEM (*n* = 4). No significant differences were observed from the control. (**B**–**D**). (**E**,**F**) Eomes-BW5147 cells were preincubated without (−) or with (+) kujiol A (40 µM) for 1 h, followed by a 2 h incubation without (−) or with (+) PMA + IM (100 nM and 1 µM) in the absence or presence of kujiol A. Representative blots of whole-cell lysates are shown (**E**). The amount of the NFATc2 protein was normalized by that of the β-actin protein. The NFATc2 protein (fold) (**F**) is represented by the mean ± SEM (*n* = 3). * *p* < 0.05 indicates significant differences between 0 and 40 µM of kujiol A. Original blots are presented in Figures S5–S12.

We examined the subcellular localization of the NFATc2 protein in BW5147 cells. In wild-type, empty vector-transfected, and Eomes-BW5147 cells, the NFATc2 protein was detected in the nuclear fraction, but not in the cytoplasmic fraction (Figure 7A–D). These results demonstrate that the NFATc2 protein is primarily localized to the nucleus, even in the absence of stimulation.

Eomes-BW5147 cells were exposed to kujiol A for 1 h and were then stimulated with PMA and IM for 2 h. Western blotting was used to detect the NFATc2 protein in whole-cell lysates. The NFATc2 protein was observed with multiple bands (Figure 7E,F). Kujiol A slightly affected the amount of the NFATc2 protein in the absence of PMA and IM stimulation (Figure 7E,F). The PMA and IM stimulation increased the electrophoretic mobility of the NFATc2 protein to some extent (Figure 7E). These results suggest that kujiol A did not markedly affect the level of the NFATc2 protein.

### 2.7. Kujiol A Suppressed the Interaction of the NFATc2 Protein with IFN-γ and IL-2 Promoters in Eomes-BW5147 Cells

**Figure 8:**
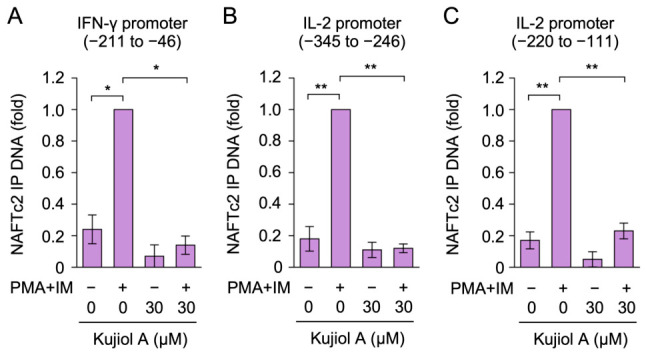
Kujiol A inhibited the NFATc2 interaction with the IFN-γ and IL-2 promoters in BW5147 cells: (**A**–**C**) Eomes-BW5147 cells were preincubated without (−) or with (+) kujiol A (30 µM) for 1 h, followed by a 6 h incubation without (−) or with (+) PMA + IM (100 nM and 1 µM) in the absence or presence of kujiol A. The amount of NFATc2-immunoprecipitated (IP) DNA (fold) is represented by the mean ± SEM (*n* = 3) in the −211 to −46 bp region of the IFN-γ promoter (**A**), the −345 to −246 bp region of the IL-2 promoter (**B**), and the −220 to −111 region of the IL-2 promoter (**C**). * *p* < 0.05 and ^**^ *p* < 0.01 indicate significant differences from PMA + IM (+).

Since the NFATc2 protein localized to the nucleus of BW5147 cells (Figure 7), a chromatin immunoprecipitation (ChIP) assay was used to investigate the effects of kujiol A on NFATc2 binding to target gene promoters. Eomes-BW5147 cells were exposed to kujiol A for 1 h and were then treated with PMA and IM for 6 h. We measured the binding efficiency of the NFATc2 protein to the IFN-γ and IL-2 promoters. The results obtained showed that the stimulation strongly augmented NFATc2 binding to the IFN-γ and IL-2 promoters (Figure 8A–C). Kujiol A markedly suppressed the increased binding efficiency of the NFATc2 protein in Eomes-BW5147 cells (Figure 8A–C). These results indicate that kujiol A prevented the NFATc2 protein from binding to the IFN-γ and IL-2 promoters.

### 2.8. An In Silico Molecular Docking Analysis Showed the Potential Binding of Kujiol A to Calcineurin and NFATc2

**Figure 9:**
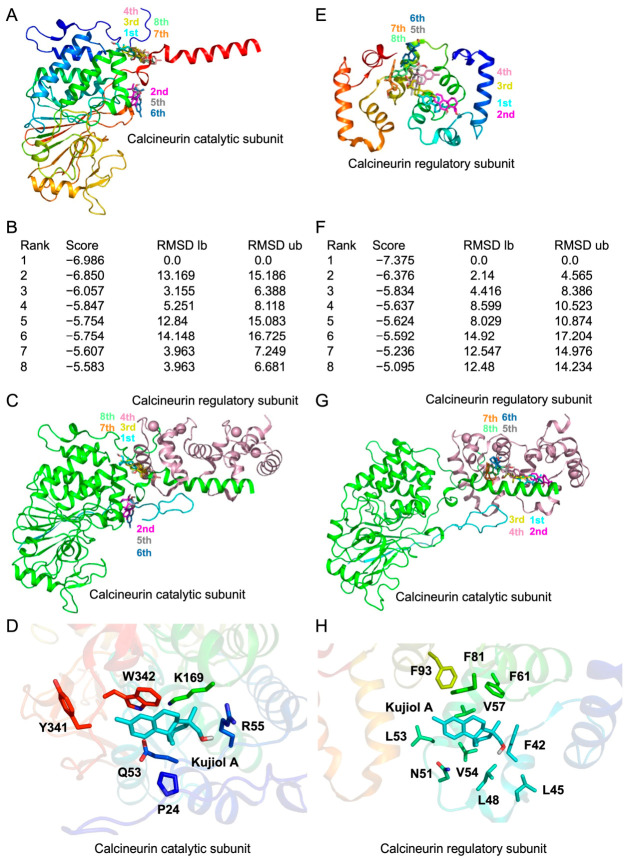
Kujiol A potentially interacted with calcineurin catalytic and regulatory subunits: (**A**–**H**) In silico docking models showing the interactions between kujiol A and the calcineurin catalytic subunit (**A**–**D**) or calcineurin regulatory subunit (**E**–**H**). Rank 1 to 8 models of the calcineurin catalytic subunit (**A**) and calcineurin regulatory subunit (**E**) are shown in different colors. Panels (**B**,**F**) show the rank, score (binding free energy; kcal/mol), root mean square deviation (RMSD) lower bound (lb), and RMSD upper bound (ub). The positions of kujiol A in ranks 1 to 8 in the calcineurin complex with the catalytic subunit (light green) and regulatory subunit (dark pink) (**C**,**G**). The positions of amino acid residues located within 4 Å of kujiol A in rank 1 models are shown (**D**,**H**). In panels (**D**,**H**), the protein structures are displayed using a rainbow color scheme (N to C: blue to red), which explains the difference in colors, and since dimers are color-coded as two molecules, their colors differ from those of monomers.

The cell-free enzyme assay using the recombinant human calcineurin catalytic and regulatory subunits showed that kujiol A inhibited dephosphorylation activity with an IC_50_ value of 42.7 µM [29]. An in silico docking analysis was conducted to assess the potential binding of kujiol A to calcineurin. Eight potential docking models were presented between kujiol A and calcineurin catalytic and regulatory subunits with scores >−5.5 kcal/mol and −5.0 kcal/mol, respectively (Figure 9A,B,E,F). The calcineurin active site is composed of D90, H92, D118, N150, H199, and H281 [41]. However, kujiol A did not directly bind to the active site of the calcineurin catalytic subunit, while it potentially interacted with several sites located on the surface of both the calcineurin catalytic subunit and the regulatory subunit (Figure 9A,E). Most of these sites, including those in the rank 1 models, were located at the interface between the catalytic and regulatory subunits of calcineurin (Figure 9C,G). In the rank 1 models, kujiol A interacted with six amino acids of the catalytic subunit and ten amino acids of the regulatory subunit (Figure 9D,H). Based on these results, kujiol A may hinder the interaction between the catalytic and regulatory subunits of calcineurin and affect its catalytic activity.

**Figure 10:**
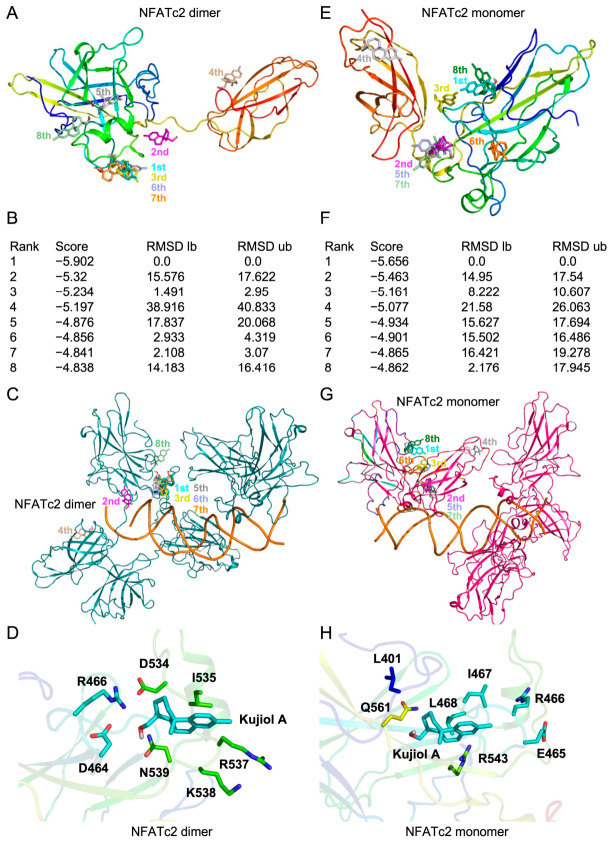
Kujiol A potentially interacted with NFATc2: (**A**–**H**) In silico docking models showing the interactions between kujiol A and the NFATc2 dimer (**A**–**D**) or NFATc2 monomer (**E**–**H**). Rank 1 to 8 models in the NFATc2 dimer (one NFATc2 molecule is presented) (**A**) and NFATc2 monomer (**E**) are shown in different colors. Panels (**B**) and (**F**) present the rank, score (binding free energy; kcal/mol), RMSD lb, and RMSD ub. The positions of kujiol A in ranks 1 to 8 in the NFATc2 dimer (dark cyan) (**C**) and NFATc2 monomer (dark pink) (**G**) complexed with DNA (orange). The positions of amino acid residues located within 4 Å of kujiol A in rank 1 models are shown (**D**,**H**). In panels (**D**,**H**), the protein structures are displayed using a rainbow color scheme (N to C: blue to red), which explains the difference in colors, and since dimers are color-coded as two molecules, their colors differ from those of monomers.

Kujiol A did not markedly affect the amount of the NFATc2 protein in the nucleus (Figure 7) but was able to prevent the binding of NFATc2 to the IFN-γ and IL-2 promoters (Figure 8). An in silico docking analysis was conducted to assess whether kujiol A binds to NFATc2 and directly inhibits its DNA-binding activity. A previous study reported that three NFATc2 molecules interacted with DNA as both a dimer and a monomer, which formed different structures [42]. Eight potential docking models with scores >−4.8 kcal/mol were observed between kujiol A and either a dimer or monomer of NFATc2 (Figure 10A,B,E,F). Kujiol A was able to bind to multiple sites of both the NFATc2 dimer and the NFATc2 monomer (Figure 10A,E). In the rank 1 models, kujiol A interacted with seven amino acids of the NFATc2 dimer and seven amino acids of the NFATc2 monomer, whereas R466 was the only amino acid common to them (Figure 10D,H), suggesting that kujiol A interacts with different sites near the dimeric and monomeric forms. These sites were not directly involved in NFATc2 binding to DNA (Figure 10C,G).

## 3. Discussion

Eomes is the lineage-specifying transcription factor that regulates epigenetic modifications to target gene loci, such as those encoding IFN-γ as a Th1 cytokine and IL-4 as a Th2 cytokine [43,44,45]. Using this T cell model, we demonstrated that kujiol A inhibited the expression of IFN-γ, IL-2, IL-4, and Fas ligand mRNAs with a similar efficiency to kujigamberol but greater than that of kujigamberoic acid A and spirolactone norditerpenoid. A comparison between kujigamberol and kujigamberoic acid A revealed that the presence of a hydroxy group was important for inhibiting cytokine expression in BW5147 cells. This result is consistent with previous findings showing that kujigamberol B, which has a hydroxy group at a different position, exhibited weaker biological activity toward HL-60 cell viability and the N-glycosylation of HUVEC than kujigamberol [27,29]. In their overall structures, kujigamberol and kujiol A share a substructure containing a hydroxy group and a benzene ring. The present results suggest that this substructure, shared between kujigamberol and kujiol A, is important for their inhibitory effects on T cell cytokine expression. However, kujigamberoic acid A was more effective at inhibiting the degranulation of RBL-2H3 cells than kujigamberol [31]. Based on these findings, compounds derived from Kuji amber, such as kujiol A, kujigamberol, and kujigamberoic acid A, may act on common and unique cellular processes and target proteins in different cell types, thereby affecting their overall inhibitory potency as MEKA [26].

Dose-dependent experiments revealed that kujiol A preferentially inhibited IFN-γ mRNA expression over IL-2 mRNA expression. We previously demonstrated that kujigamberol preferentially inhibited IL-2 mRNA expression over IFN-γ mRNA expression in Eomes-BW5147 cells [28]. Collectively, the present results and previous findings revealed that kujiol A and kujigamberol showed distinct inhibitory profiles for IFN-γ and IL-2 expression. The different inhibitory profiles of kujigamberol and kujiol A were also observed for the transcriptional activity of NFAT-Luc and NF-κB-Luc in 293T cells. While kujigamberol did not affect NF-κB-Luc activity [28], the present study showed that kujiol A inhibited NF-κB-Luc activity. Kujiol A markedly suppressed NFAT-dependent transcriptional activity, as previously observed with kujigamberol [28]. Therefore, kujiol A appears to inhibit NFAT-Luc and NF-κB-Luc activities, while kujigamberol may only suppress NFAT-Luc activity. The IFN-γ and IL-2 promoters contain binding sites for NFAT and NF-κB [46,47,48,49]. Consistent with these initial findings, potential NFAT sites have been identified in the murine IFN-γ and IL-2 promoters [28]. The calcineurin inhibitor FK506 potently diminished the expression of both IFN-γ and IL-2 mRNA in Eomes-BW5147 cells [50,51]. In contrast, the IκB kinase inhibitor IKK-16 only slightly reduced IFN-γ mRNA expression at a concentration that almost completely diminished IL-2 mRNA expression in Eomes-BW5147 cells [51], suggesting that NF-κB is auxiliary for IFN-γ expression. Therefore, kujiol A and kujigamberol may primarily prevent NFAT-dependent transcriptional activity, thereby blocking IFN-γ and IL-2 mRNA expression.

The NFAT family consists of five members, four of which are primarily regulated by Ca^2+^ signaling [35,36,37]. T cells express multiple NFAT proteins, including NFATc2 [21,23]. In addition to IFN-γ and IL-2 [46,47,48,49], NFAT binding sites are present in the IL-4 and Fas ligand promoters [52,53]. NFATc2 is critical for the transcription of IFN-γ, IL-4, and Fas ligand [38,39,40], whereas the transcription of IL-2 is regulated by both NFATc1 and NFATc2 [54]. Previous studies using mass spectrometry revealed that NFATc2 was phosphorylated at 14 conserved phosphoserine residues, 13 of which were dephosphorylated upon stimulation [55]. NFAT proteins generally have regulatory regions with serine-rich sequences that are phosphorylated by numerous protein kinases [56,57]. Consequently, the NFATc2 protein becomes hyperphosphorylated in the cytoplasm, and the Ca^2+^-dependent activation of calcineurin then dephosphorylates the NFATc2 protein, allowing its translocation to the nucleus [21,22,23,24]. The IM stimulation of CTLL-2 cells and the PMA and IM stimulation of BW5147 cells slightly increased the electrophoretic mobility of the NFATc2 protein, suggesting its dephosphorylation. Unlike primary T cells, the NFATc2 protein appears to have a different phosphorylated/dephosphorylated status in BW5147 cells and CTLL-2 cells.

Calcineurin is responsible for the dephosphorylation of NFAT family proteins, including NFATc2 [18,19,20]. In Eomes-BW5147 cells, the electrophoretic mobility of the NFATc2 protein was reduced by FK506 irrespective of the PMA and IM stimulation [28]. In contrast, kujiol A did not markedly affect the electrophoretic mobility of the NFATc2 protein under unstimulated or stimulated conditions in Eomes-BW5147 cells and CTLL-2 cells, similar to kujigamberol [28]. Previous enzyme assays showed that kujiol A inhibited calcineurin with an IC_50_ value of 42.7 µM [29], whereas kujigamberol was inactive (>200 µM) [25]. In silico docking models revealed that kujiol A potentially binds calcineurin catalytic and regulatory subunits and hinders their interaction. However, the effects of kujiol A on calcineurin and its substrate NFATc2 in cells were weaker than those of FK506 [28]. Our results do not exclude the possibility that kujiol A inhibits other Ca^2+^-dependent processes. Due to the presence of many potential phosphorylation sites, it is currently unclear whether stimulation with PMA or IM or treatment with kujigamberol or kujiol A affected the specific phosphorylation and dephosphorylation sites of the NFATc2 protein in BW5147 cells and CTLL-2 cells.

Previous studies demonstrated that PMA and IM stimulation or T cell receptor stimulation strongly promoted the NFATc2 protein interaction with the IFN-γ and IL-2 promoters [58,59]. Consistent with these findings, we showed that PMA and IM stimulation strongly enhanced the NFATc2 protein interaction with the IFN-γ and IL-2 promoters in Eomes-BW5147 cells [28,50,51]. We found that the NFATc2 protein localized to the nucleus of unstimulated BW5147 cells and CTLL-2 cells. This unexpected result is consistent with previous findings showing that a large amount of the NFATc2 protein was constitutively localized to the nucleus in rat vascular smooth muscle cells and human breast cancer cell lines [60,61] and also that a certain amount of the NFATc2 protein was present in the nucleus of mouse T regulatory T cells without any stimulation [62]. Therefore, depending on the cellular context, it appears that the NFATc2 protein is present in the nucleus in an inactive form and becomes transcriptionally active after stimulation. The ChIP assay demonstrated that kujiol A prevented the NFATc2 protein from interacting with the IFN-γ and IL-2 promoters in Eomes-BW5147 cells stimulated with PMA and IM. This result is consistent with luciferase reporter assays showing that kujiol A inhibited NFAT-Luc activity. The results of the in silico docking assay suggest that kujiol A did not directly affect the DNA-binding activity of NFATc2 but rather appeared to prevent the interaction of the catalytic subunit and regulatory subunit of calcineurin. Further studies are needed to confirm whether kujiol A binds to NFATc2 or calcineurin and inhibits their biological activities in cells, as well as to clarify the primary mechanisms by which kujiol A targets the Ca^2+^–calcineurin–NFAT pathway.

## 4. Materials and Methods

### 4.1. Cells

BW5147 cells (murine T cell lymphoma; JCRB9002) were distributed by the JCRB Cell Bank of the National Institutes of Biomedical Innovation, Health, and Nutrition (Osaka, Japan). BW5147 cells stably transfected with an expression vector encoding FLAG-Eomes (Eomes #2 BW5147 cells) or an empty vector (Control #2 BW5147 cells) were generated in our previous studies [50,51]. 293T cells (human embryonic kidney cell; RCB2202) and CTLL-2 cells (murine cytotoxic T cell line; RCB0637) were distributed by the RIKEN BioResource Research Center (Tsukuba, Japan). RPMI 1640 medium and DMEM medium were supplemented with heat-inactivated fetal calf serum (Nichirei Bioscience, Tokyo, Japan) and the penicillin–streptomycin antibiotic mixture (Nacalai Tesque, Kyoto, Japan). BW5147 cells and 293T cells were subcultured with RPMI 1640 medium and DMEM medium, respectively. CTLL-2 cells were subcultured with RPMI 1640 medium supplemented with recombinant human IL-2 (100 U/mL) (PeproTech, Cranbury, NJ, USA).

### 4.2. Reagents

Kujigamberol (15,20-dinor-5,7,9-labdatrien-18-ol), kujigamberoic acid A (15,20-dinor-5,7,9-labdatrien-18-oic acid), kujiol A (13-methyl-8,11,13-podocarpatrien-19-ol), and spirolactone norditerpenoid [(4R*, 5S*, 8R*, 9R*, 10S*)-14,15,16,19,-tetranor-labdatrien-13-ol] were prepared as previously described [25,29,30,31]. PMA was obtained from Wako Pure Chemical Industries (Osaka, Japan). IM was purchased from Merck Millipore (Darmstadt, Germany). Dimethyl sulfoxide (DMSO) was used as a vehicle to prepare stock solutions of kujigamberol (20 mM), kujigamberoic acid A (20 mM), kujiol A (20 mM), and spirolactone norditerpenoid (20 mM). These stock solutions were diluted with culture medium to achieve a final DMSO concentration of less than 0.25%. Concentrations that did not reduce cell viability were used for subsequent experiments.

### 4.3. Antibodies

The antibodies used in this study were as follows: primary antibodies specific to NFATc2 (sc-7296; Santa Cruz Biotechnology, Dallas, TX, USA), lamin A/C (sc-376248; Santa Cruz Biotechnology), GAPDH (sc-32233; Santa Cruz Biotechnology), β-actin (A5441; Sigma-Aldrich, St. Louis, MO, USA), and anti-mouse IgG (H + L) antibody, peroxidase-conjugated (115-035-146; Jackson ImmunoResearch Laboratories, West Grove, PA, USA).

### 4.4. Plasmid Vectors

Reporter vectors encoding NF-κB firefly luciferase (NF-κB-Luc), AP-1 firefly luciferase (AP-1-Luc), and NFAT firefly luciferase (NFAT-Luc) (all kind gifts from Prof. Ralph C. Budd) and reporter vectors possessing a herpes simplex virus thymidine kinase promoter-driven *Renilla* luciferase and a cytomegalovirus promoter-driven *Renilla* luciferase were used in luciferase reporter assays.

### 4.5. Cell Viability Assay

BW5147 cells (5 × 10^4^ cells/well), CTLL-2 cells (5 × 10^4^ cells/well), and 293T cells (2 × 10^4^ cells/well) were preincubated in 96-well cell culture plates overnight prior to compound treatment. MTT (Nacalai Tesque, Kyoto, Japan) was used to evaluate cell viability as previously described [28]. Three biological replicates were used in each experiment. The average of three replicates was determined, and the control values were set to 100% in each experiment.

### 4.6. RT-qPCR

BW5147 cells (6 × 10^5^ cells/dish) and CTLL-2 cells (5 × 10^5^ cells/dish) were preincubated in 35-mm cell culture dishes one day prior to compound treatment. The preparation of total RNA, the synthesis of complementary DNA, and real-time PCR were conducted as previously described [28]. Primers used for real-time PCR to evaluate IFN-γ, IL-2, IL-4, and Fas ligand expression were previously described [63,64,65]. Primer-specific standard curves were used to quantitate the initial amount of DNA. The amount of target DNA in each sample was normalized to that of β-actin. The control values were set to 100% in each experiment.

### 4.7. Reporter Assay

293T cells (5 × 10^4^ cells/well) were preincubated in 24-well cell culture plates one day prior to compound treatment. Cells were transfected with firefly and *Renilla* luciferase reporter vectors using Polyethyleneimine Max (PEI MAX^®^) (Polysciences, Warrington, PA, USA). The preparation of cytosolic fractions and firefly and analyses using *Renilla* luciferase assays were performed as previously described [28]. Three biological replicates were used in each experiment. The firefly luciferase activity of each replicate was normalized to the *Renilla* luciferase activity. The average of three replicates was determined, and the control values were set to 100% in each experiment.

### 4.8. Western Blotting

BW5147 cells (6 × 10^5^ cells/dish) and CTLL-2 cells (5 × 10^5^ cells/dish) were preincubated in 35 mm cell culture dishes one day prior to compound treatment. The preparation of whole-cell lysates and nuclear and cytoplasmic fractions for SDS–polyacrylamide gel electrophoresis and Western blotting was conducted as previously described [28,50]. Blots were reprobed with loading control antibodies. The amount of target proteins in each sample was normalized to that of the loading controls. Control values in each experiment were set to 1-fold. For cell fractionation assays, total protein levels in each experiment were set to 100%.

### 4.9. ChIP Assay

BW5147 cells (5 × 10^6^ cells/dish) were preincubated in 100 mm cell culture dishes one day prior to compound treatment. Cells were fixed, collected, and then sonicated to shear DNA, followed by the preparation of input and immunoprecipitated DNA as previously described [28,50]. The primers used in real-time PCR to amplify IFN-γ and IL-2 promoter regions were previously described [58,59,66]. Primer-specific standard curves were used to quantitate the initial amount of DNA. The amount of immunoprecipitated DNA in each sample was normalized to the amount of input DNA. Control values were set to 1-fold in each experiment.

### 4.10. Statistical Analysis

Experiments, including cell passages and compound treatments, were performed independently on different days. Data are presented as the mean ± SEM from three or more independent experiments. For normalized assays, the corresponding control value was set to 100% or 1-fold within each independent experiment, and the normalized values were used for summary statistics and statistical analysis. Normality was assessed descriptively using the Shapiro–Wilk test. As no consistent evidence of departure from normality was observed across the datasets, parametric tests were applied consistently. In many normalized datasets, the corresponding control group was fixed at 100% or 1-fold, resulting in zero variance by definition. For this reason, the primary statistical analysis focused on pre-specified comparisons between each treatment group and its corresponding normalized control group. Direct control-versus-treatment comparisons were performed using Welch’s *t*-tests. When multiple comparisons were made within a figure panel, *p* values were adjusted using the Holm method. A *p* value of less than 0.05 after adjustment was considered statistically significant. KaleidaGraph software version 4.5.1 (Hulinks, Tokyo, Japan) and Python software version 3.12.13 (https://colab.research.google.com/ accessed on 1 April 2026) were used for graph drawing and statistical analyses.

### 4.11. In Silico Docking Analysis

An in silico docking analysis was conducted using AutoDock Vina, version 1.1.2 [67,68]. The human calcineurin catalytic subunit and regulatory subunit (PDB ID: 4F0Z) and human NFATc2 dimer and monomer forms (PDB ID: 2O93) complexed with kujiol A were evaluated. Center coordinates (x, y, z) and box sizes were (30, 30, 70) and 100 × 100 × 100 for the calcineurin catalytic subunit, (60, 60, 100) and 100 × 80 × 80 for the calcineurin regulatory subunit, and (50, 50, 50) and 100 × 100 × 100 for the NFATc2 dimer and monomer forms, respectively.

## 5. Conclusions

The present study demonstrated that kujiol A suppressed the mRNA expression of T cell cytokines. Kujiol A potently inhibited NFAT transcriptional activity and interfered with NFATc2 DNA-binding activity. These inhibitory activities were similar to those of kujigamberol. However, unlike kujigamberol, kujiol A reduced NF-κB transcriptional activity and preferentially inhibited IFN-γ mRNA expression over IL-2 mRNA expression. Based on these results, kujigamberol-related compounds have multiple molecular targets in cells, and slight substructural differences have an impact on their inhibitory profiles for cellular targets. We previously demonstrated that the activity of kujigamberol was five-fold higher than that of the clinical drug mometasone furoate in guinea pigs with nasal congestion [26]. This suggests that even kujiol A, which has a different structure, potentially exhibits high activity at the animal level. In the future, in vivo validation of kujiol A is necessary to discuss its therapeutic potential. In conclusion, the present study demonstrated the potential of kujiol A as an immunosuppressant candidate to block T cell cytokine expression.

## Data Availability

Data will be made available upon reasonable request.

## Supplementary Materials

The following supporting information can be downloaded at: https://www.mdpi.com/article/10.3390/molecules31101613/s1, Figure S1: Original blots in Figure 6A; Figure S2: Original blots in Figure 6B (Exp. 1); Figure S3: Original blots in Figure 6B (Exp. 2); Figure S4: Original blots in Figure 6B (Exp. 3); Figure S5: Original blots in Figure 7A; Figure S6: Original blots in Figure 7B; Figure S7: Original blots in Figure 7C; Figure S8: Original blots in Figure 7D; Figure S9: Original blots in Figure 7E; Figure S10: Original blots in Figure 7F (Exp. 1); Figure S11: Original blots in Figure 7F (Exp. 2); Figure S12: Original blots in Figure 7F (Exp. 3).

## Abbreviations

The following abbreviations are used in this manuscript:

IFN-γ	Interferon-γ
Th1	T helper type 1
IL	Interleukin
Th2	T helper type 2
AP-1	Activated protein-1
NF-κB	Nuclear factor κB
NFAT	Nuclear factor of activated T cells
HUVEC	Human umbilical vein endothelial cells
Eomes	Eomesodermin
PMA	Phorbol 12-myristate 13-acetate
IM	Ionomycin
SEM	Standard error of the mean
IC_50_	50% inhibitory concentration
RMSD	Root mean square deviation
lb	Lower bound
ub	Upper bound
RT-qPCR	Reverse transcription quantitative polymerase chain reaction
GAPDH	Glyceraldehyde-3-phosphate dehydrogenase
ChIP	Chromatin immunoprecipitation
MEKA	Methanol extract of Kuji amber
