# Supplementary material for: Kujiol A Inhibits Interferon-γ and Interleukin-2 Expression and the NFATc2 Interaction with Their Promoters in T Cells
Source: Molecules. 2026 May 11;31(10):1613. doi: 10.3390/molecules31101613 (PMC13210300; doi:10.3390/molecules31101613)

Figure S1: Original blots in Figure 6A

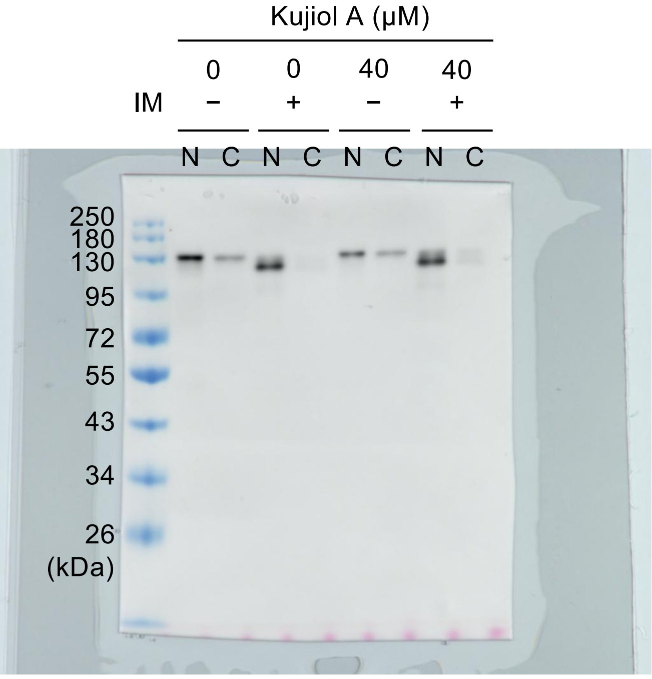

WB: NFATc2

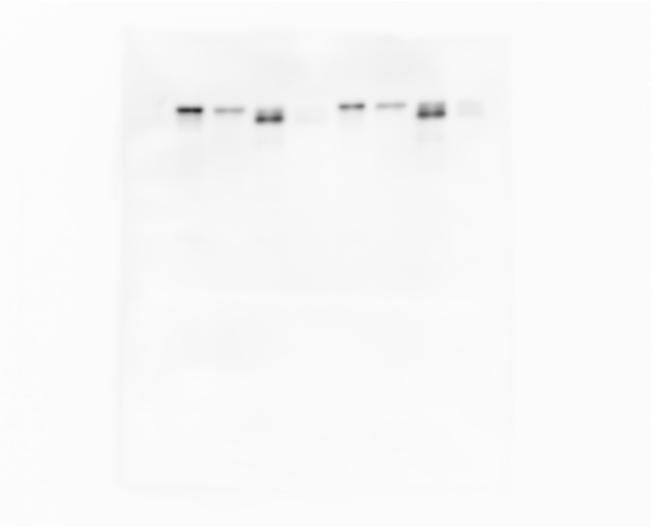

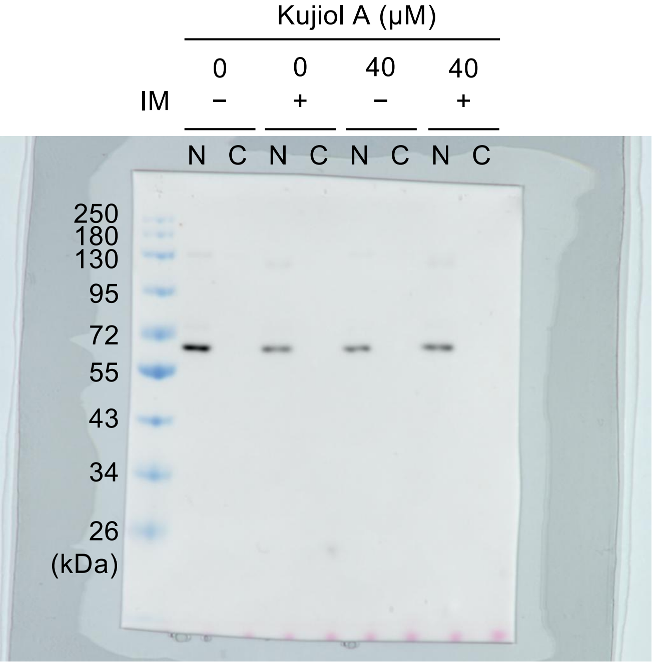

WB: Lamin A/C (reprobed)

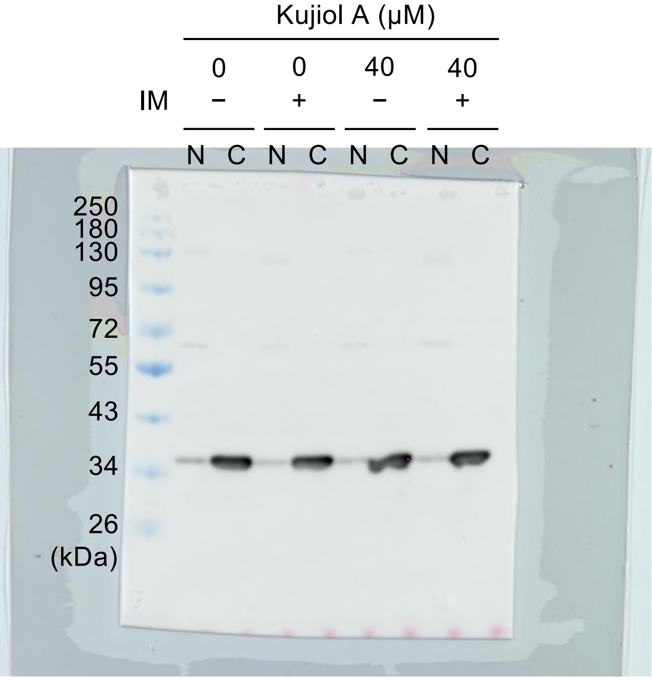

WB: GAPDH (reprobed)

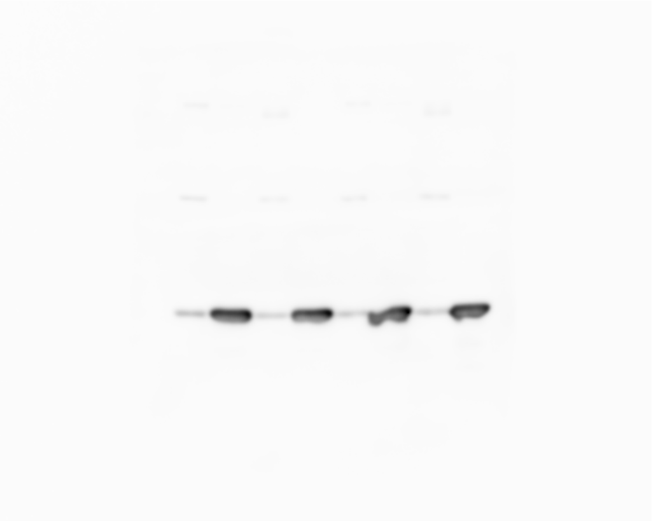

Figure S2: Original blots in Figure 6B (Exp. 1)

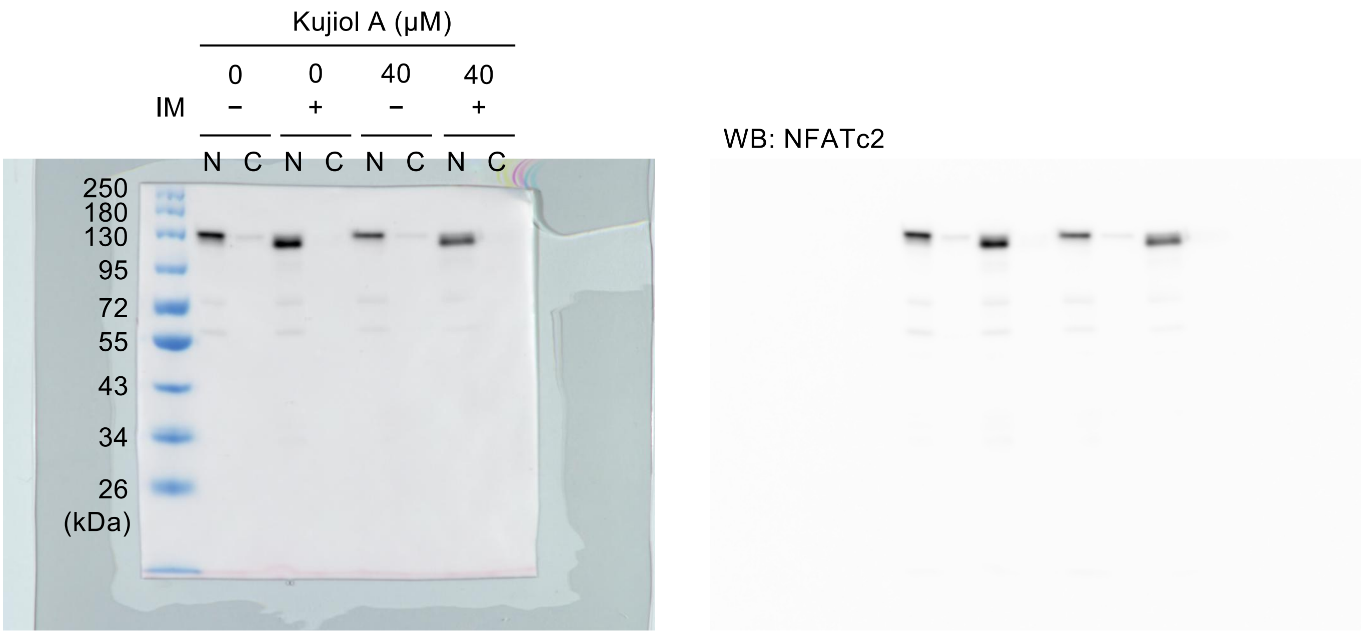

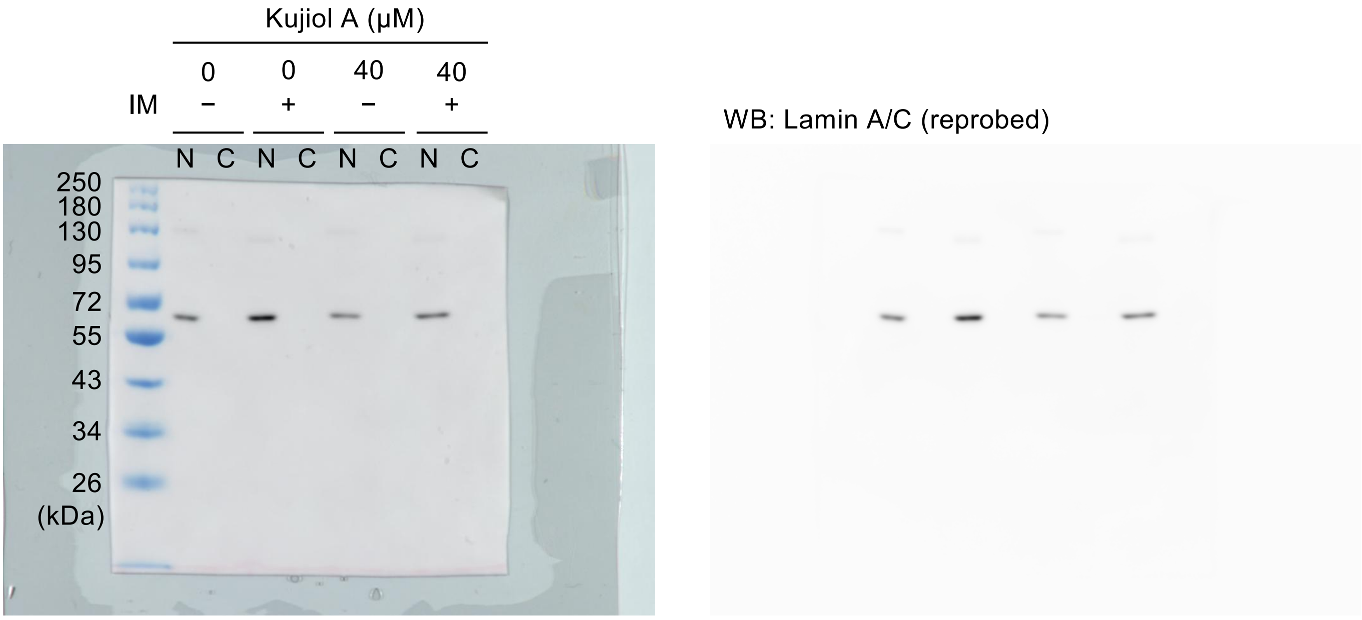

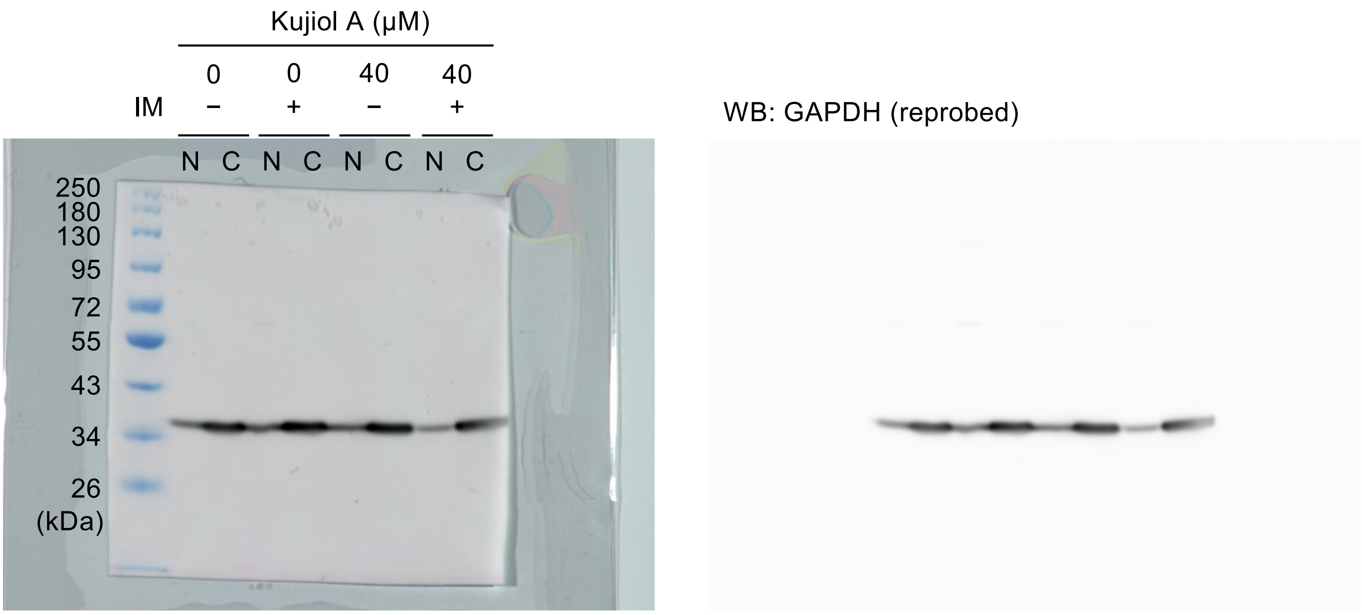

Figure S3: Original blots in Figure 6B (Exp. 2)

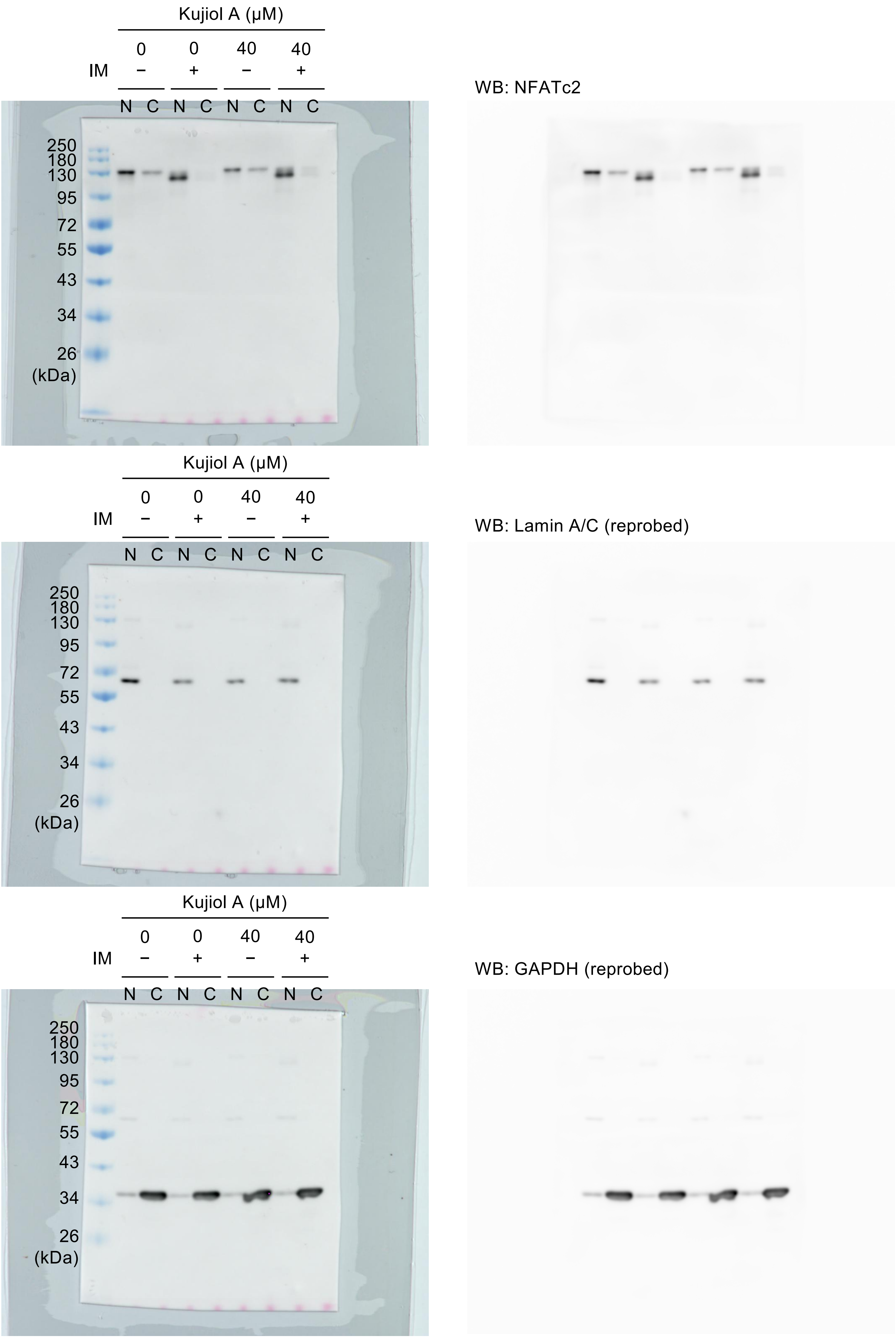

Figure S4: Original blots in Figure 6B (Exp. 3)

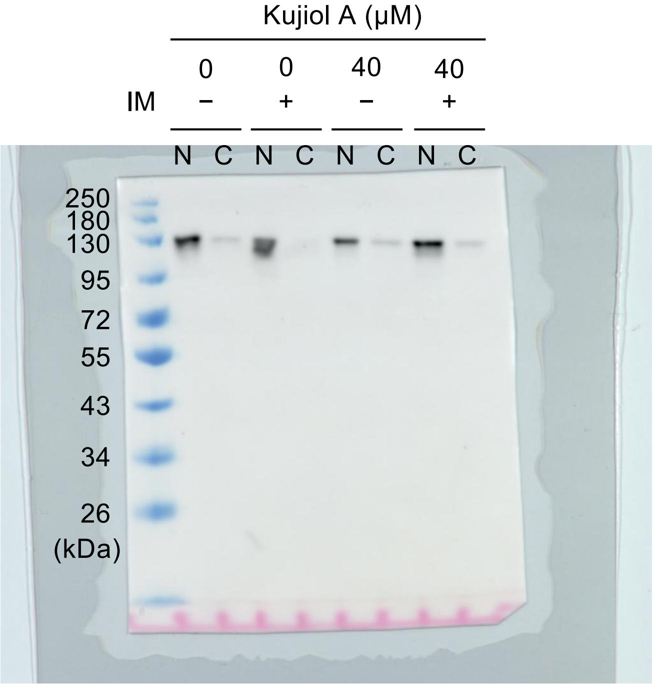

WB: NFATc2

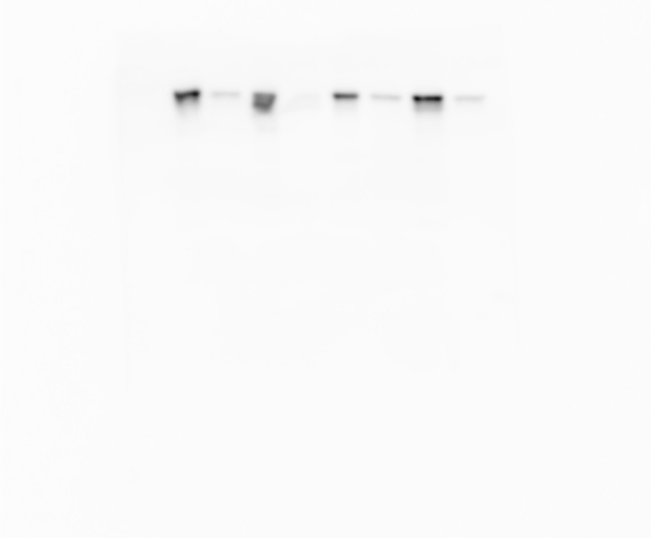

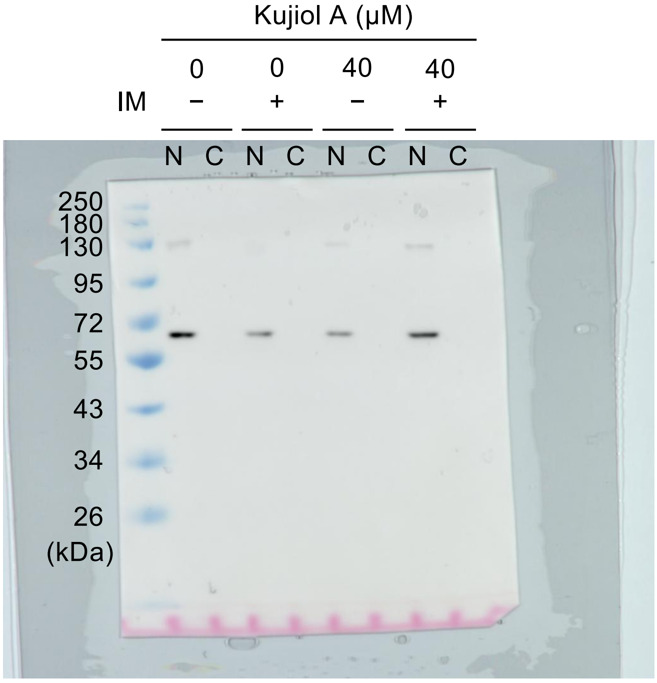

WB: Lamin A/C (reprobed)

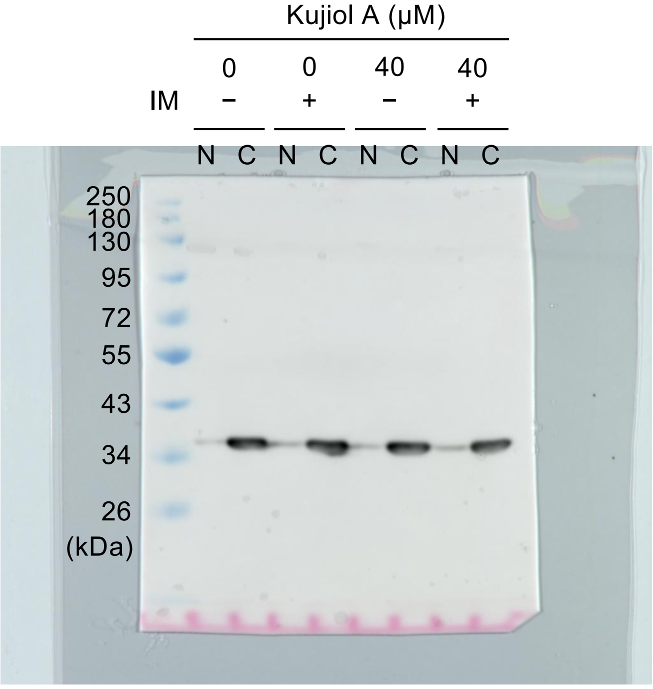

WB: GAPDH (reprobed)

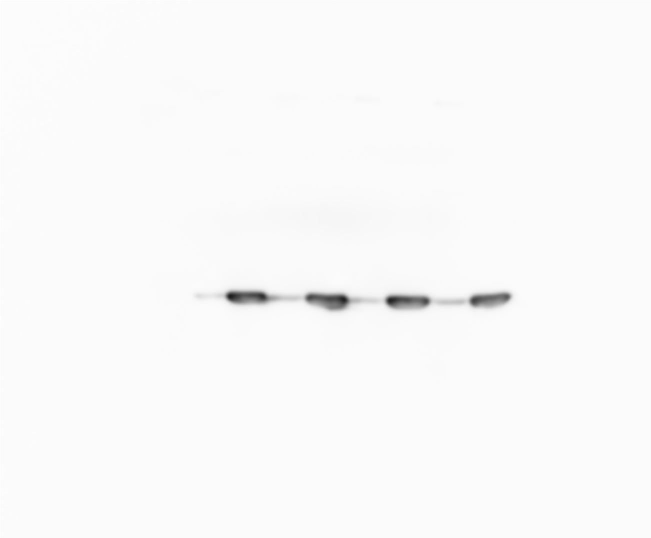

Figure S5: Original blots in Figure 7A

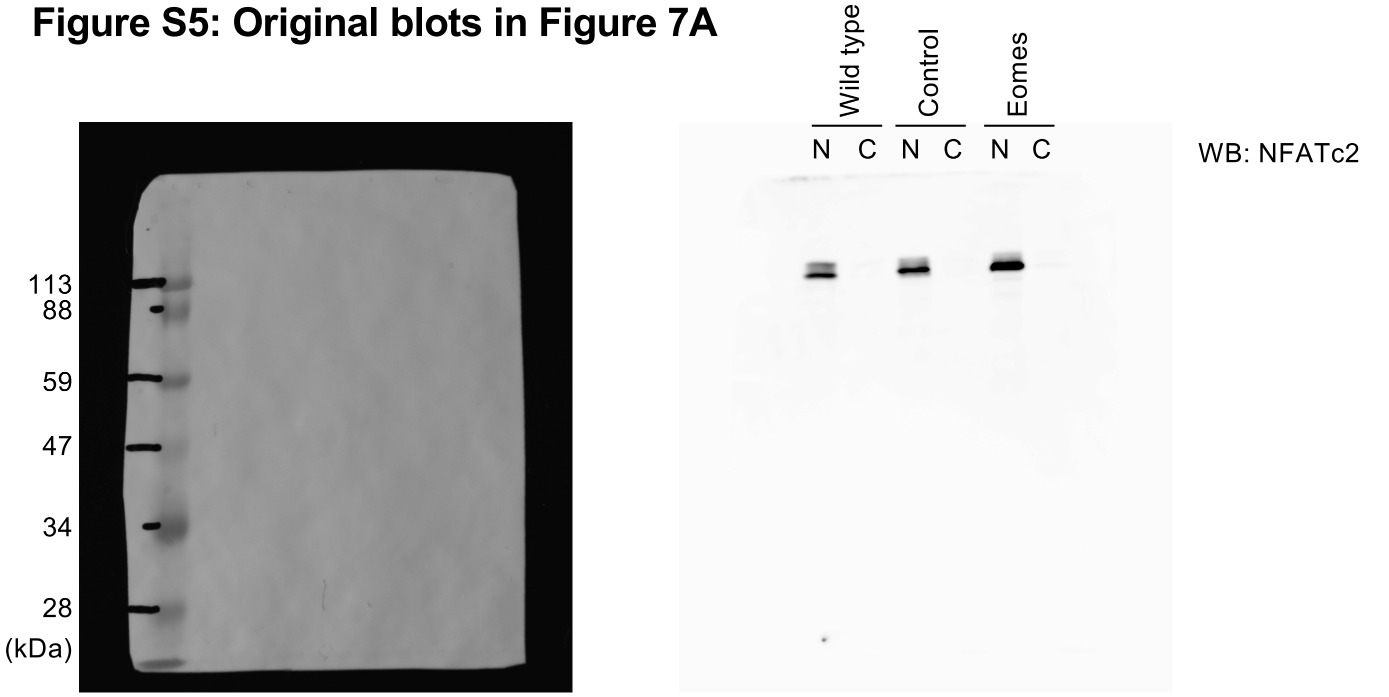

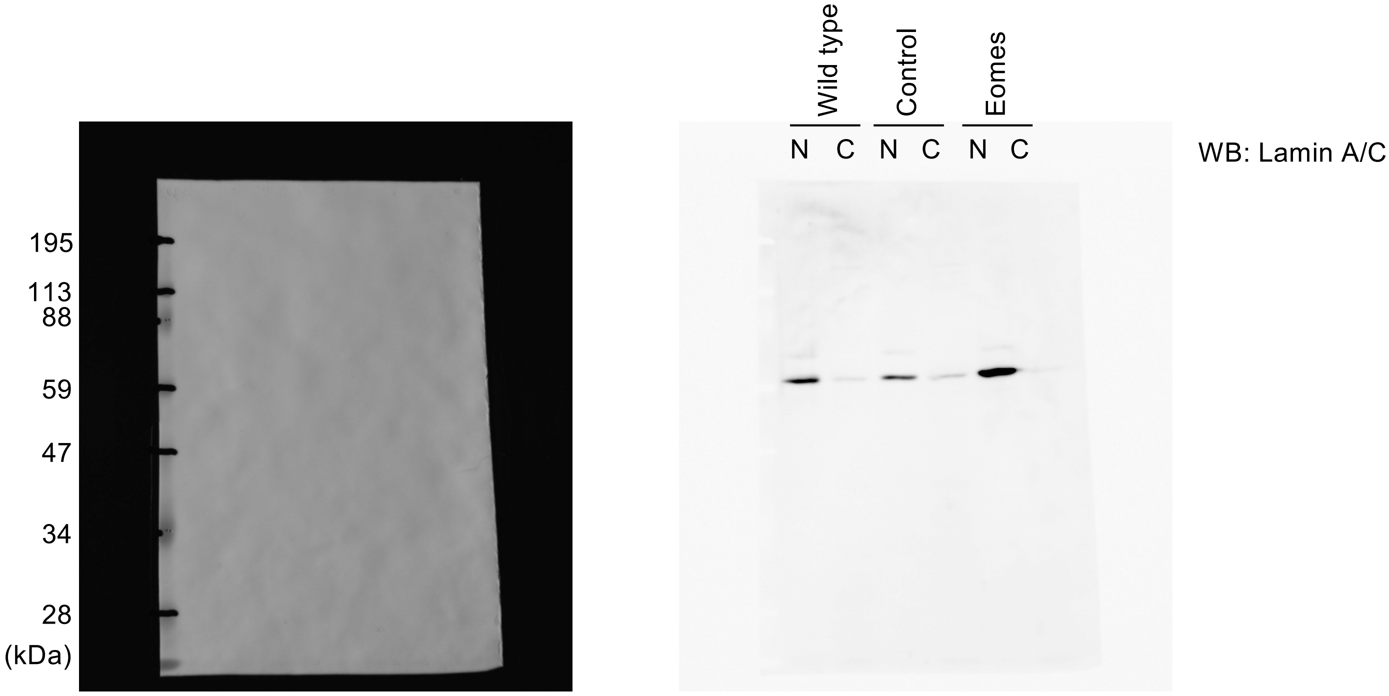

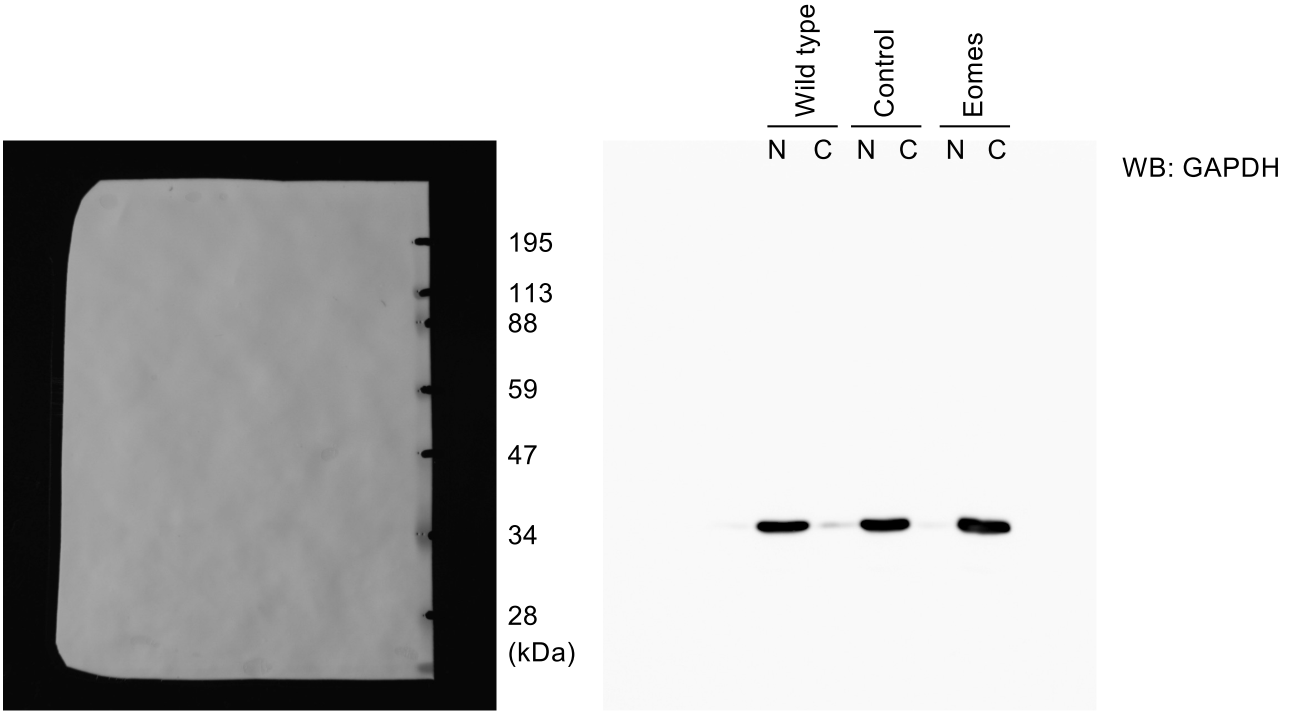

Figure S6: Original blots in Figure 7B

Exp. 1

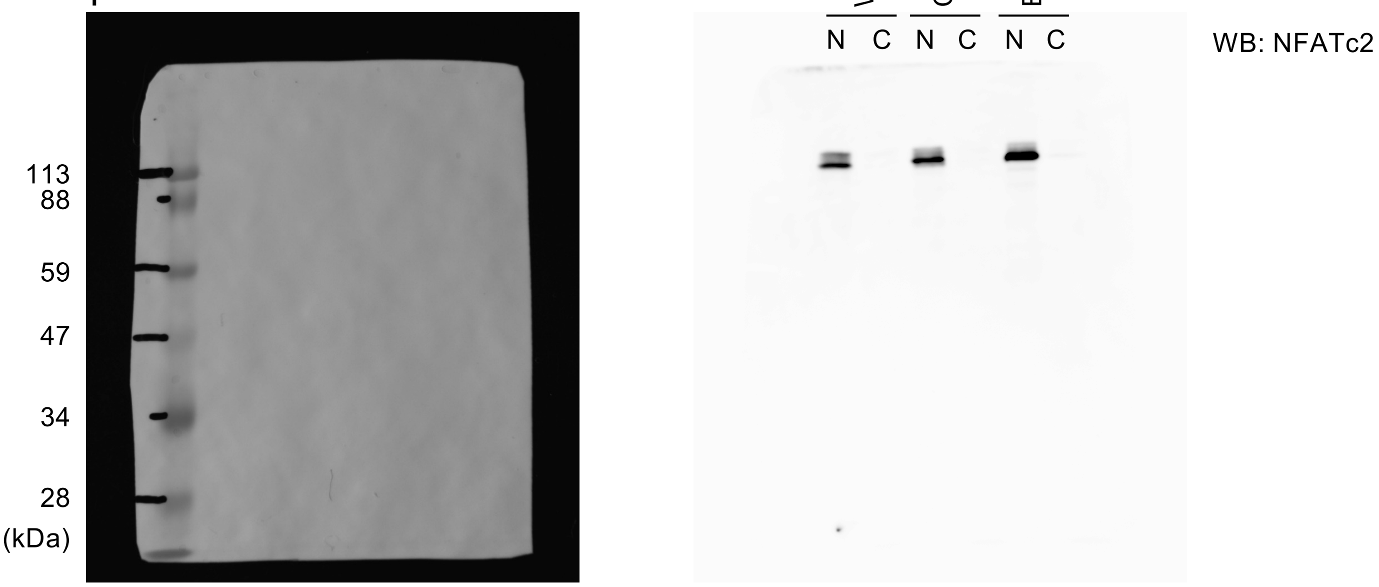

Exp. 2

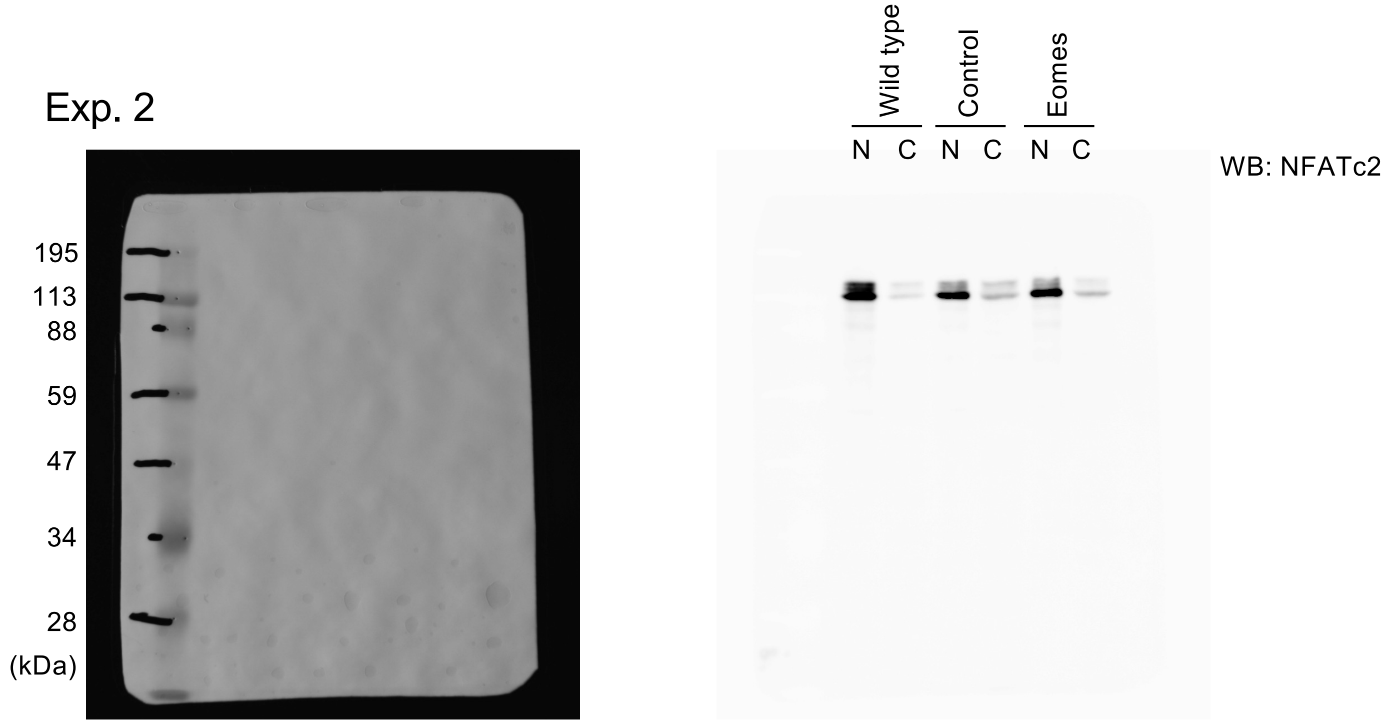

Exp. 3

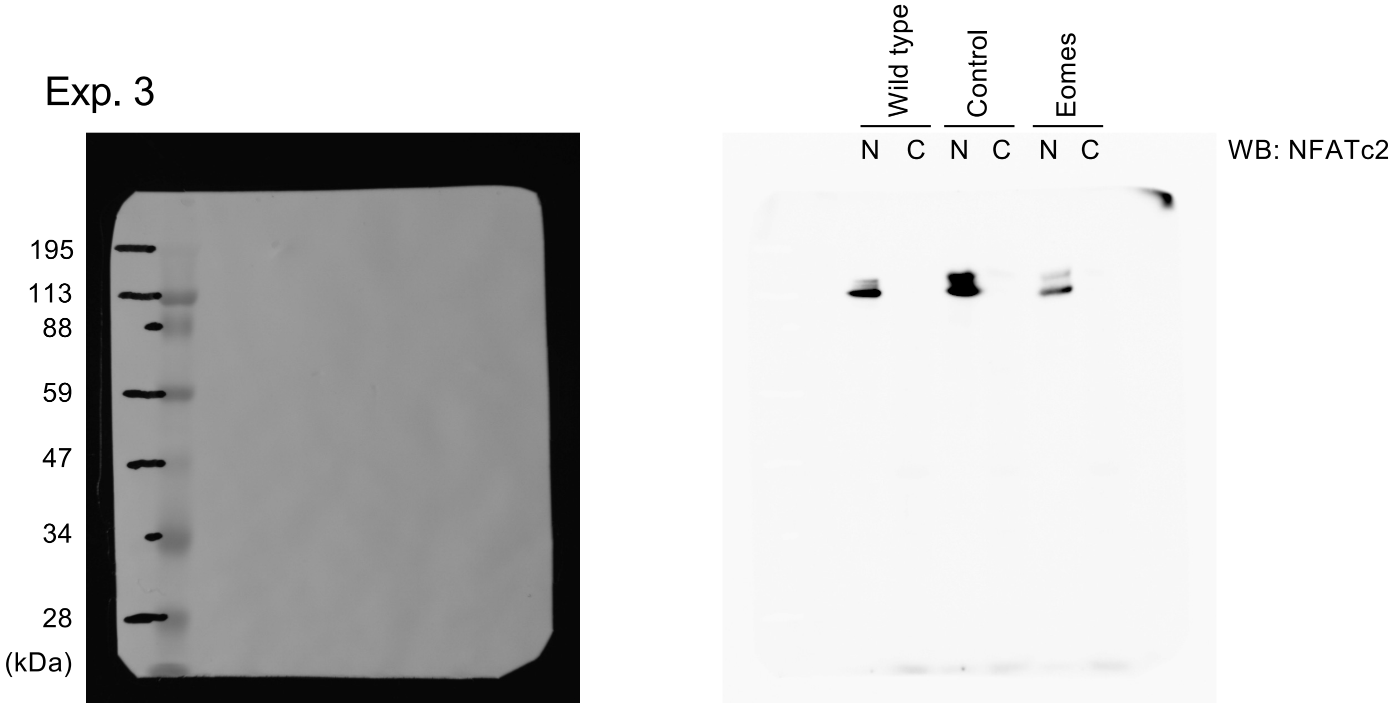

Figure S6: Original blots in Figure 7B (continued)

Exp. 4

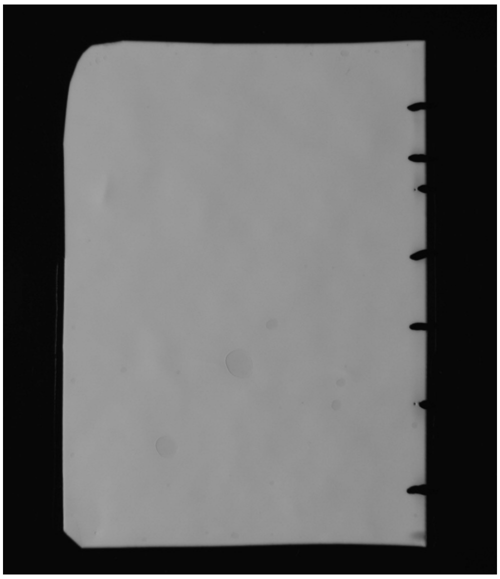

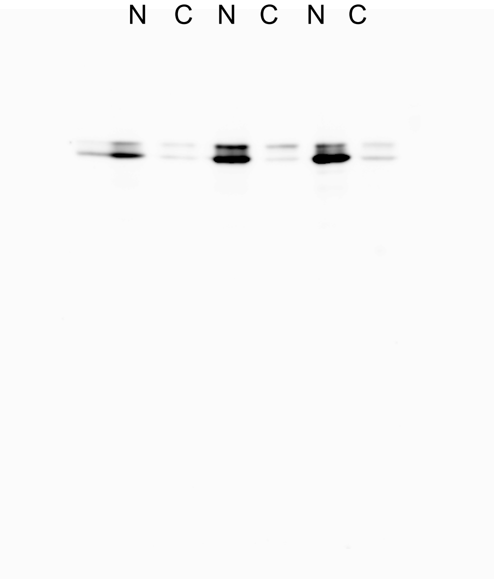

Figure S7: Original blots in Figure 7C

Exp. 1

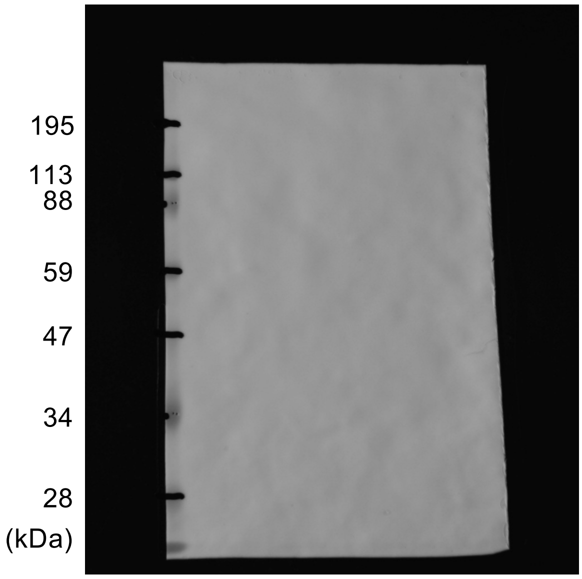

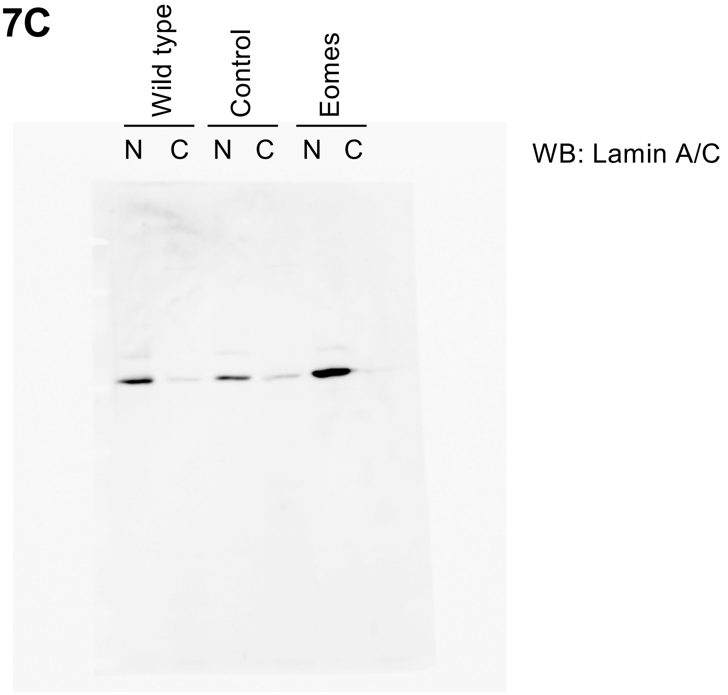

Exp. 2

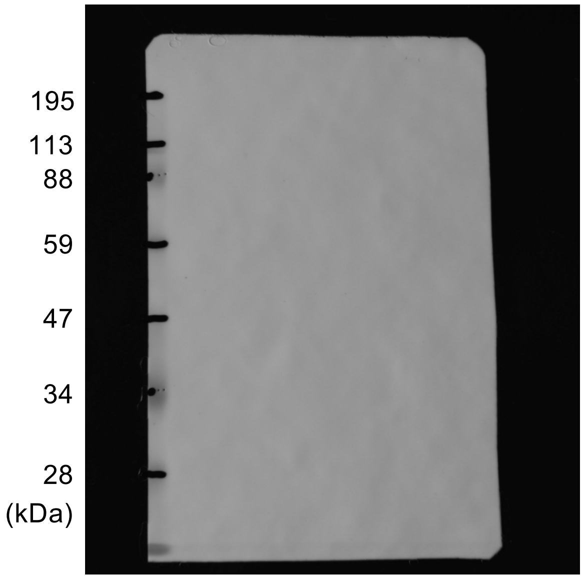

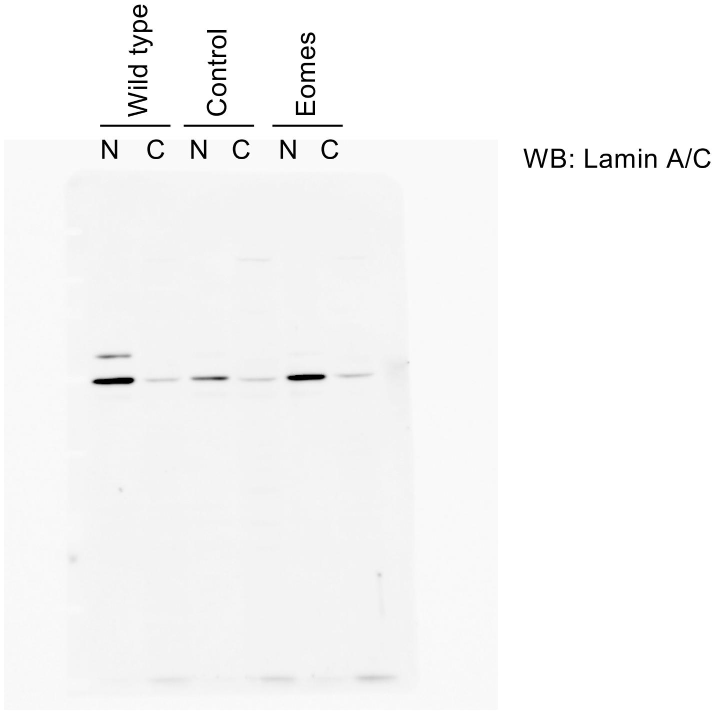

Exp. 3

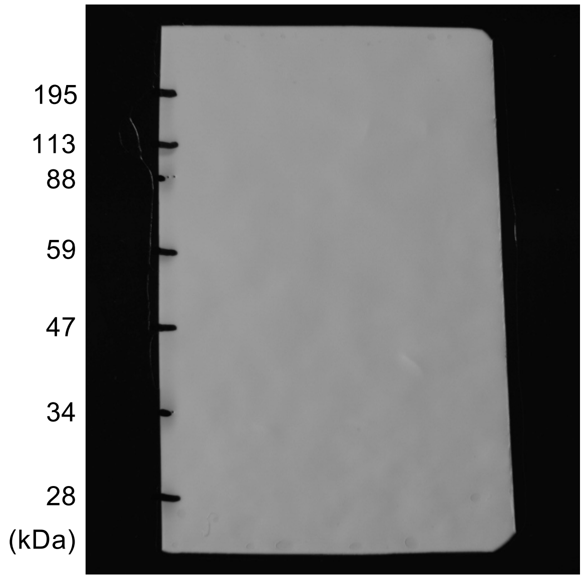

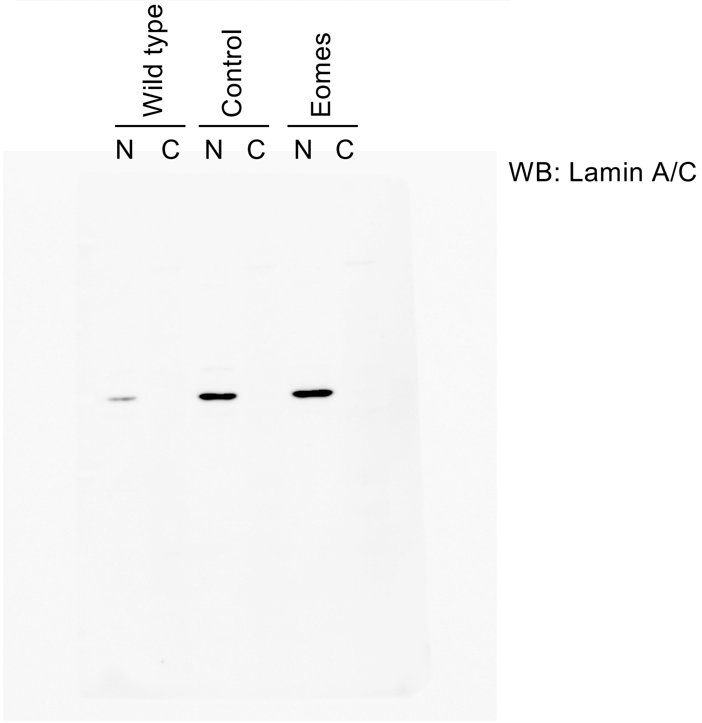

Figure S7: Original blots in Figure 7C (continued)

Exp. 4

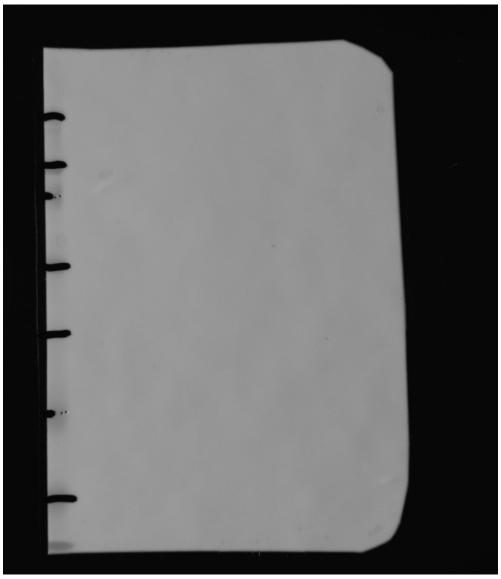

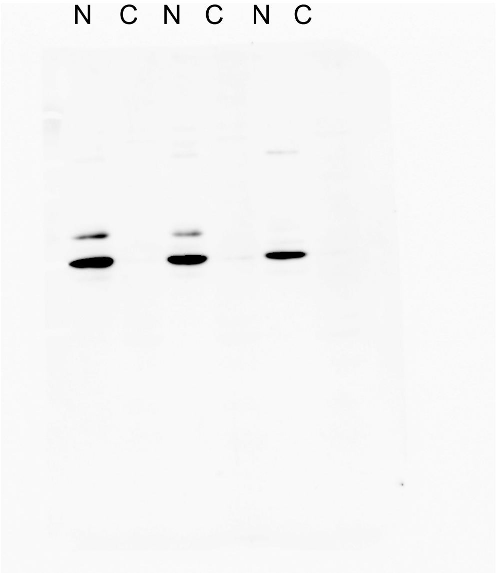

Figure S8: Original blots in Figure 7D

Exp. 1

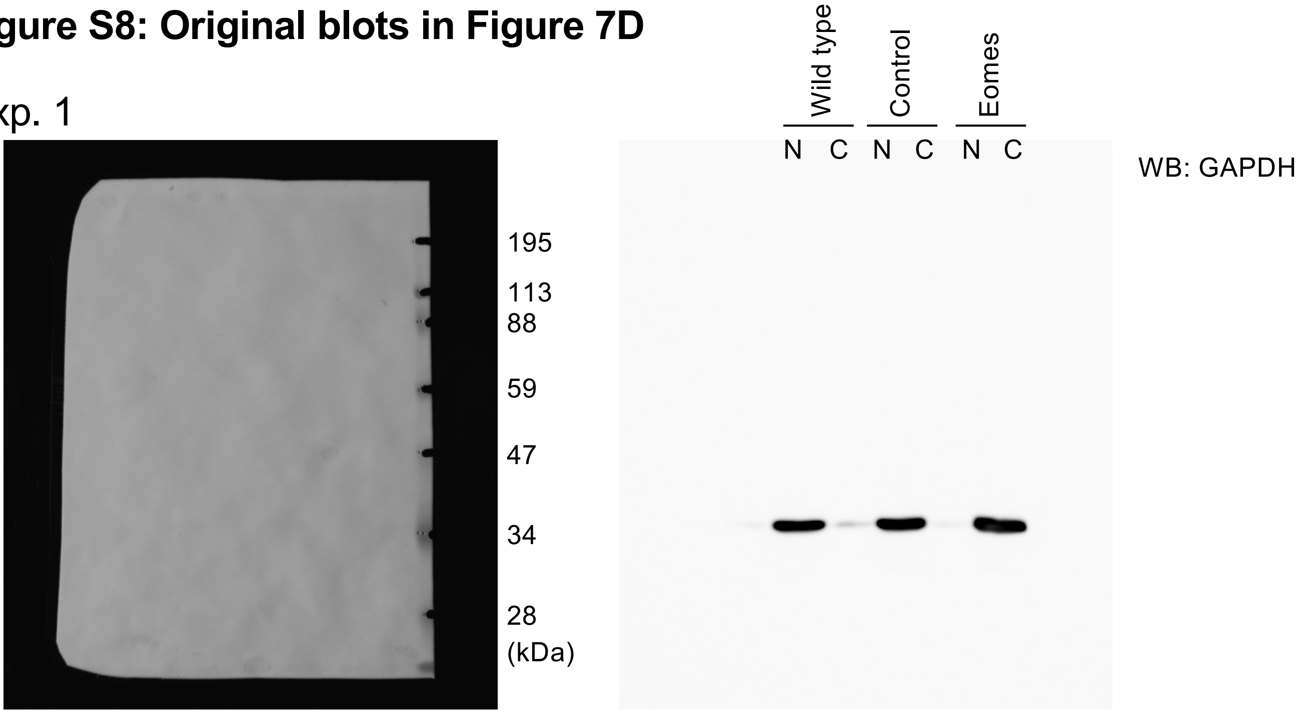

Exp. 2

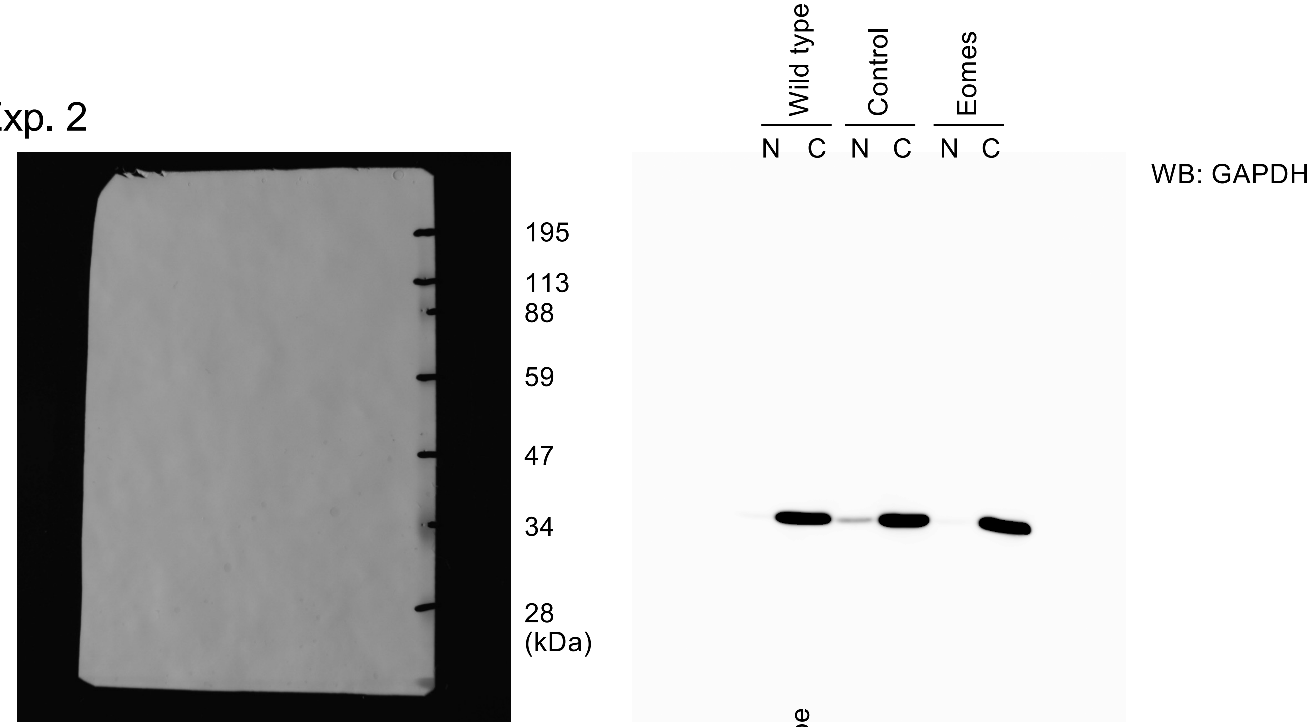

Exp. 3

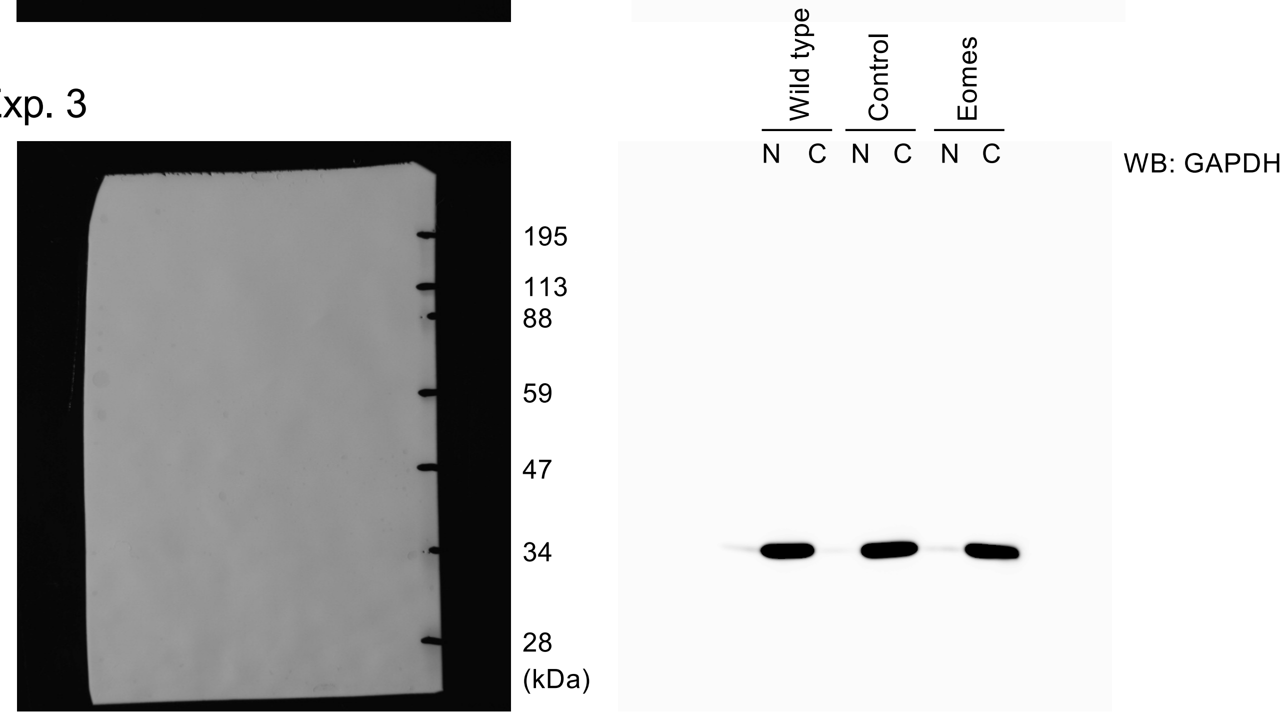

Figure S8: Original blots in Figure 7D (continued)

Exp. 4

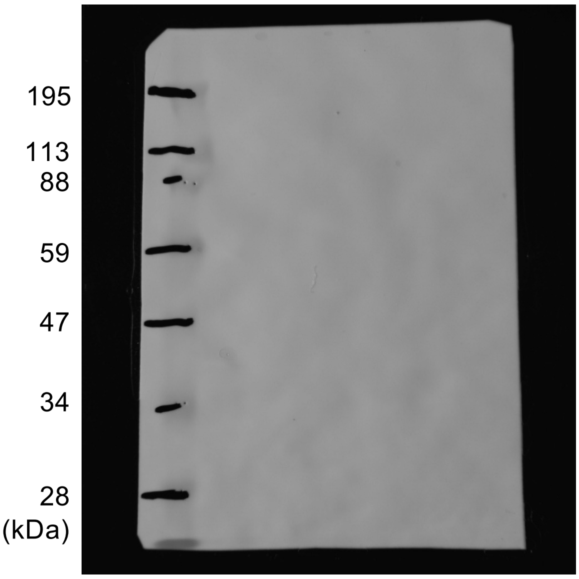

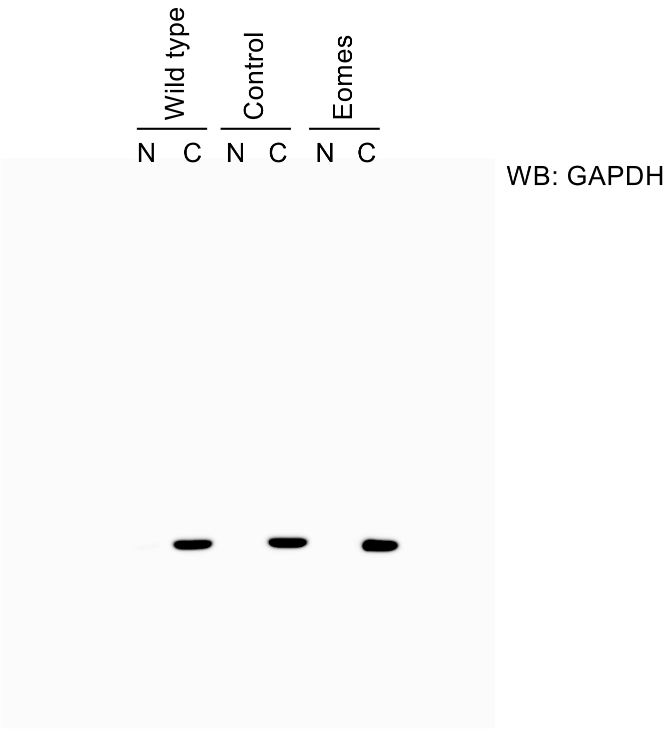

Figure S9: Original blots in Figure 7E

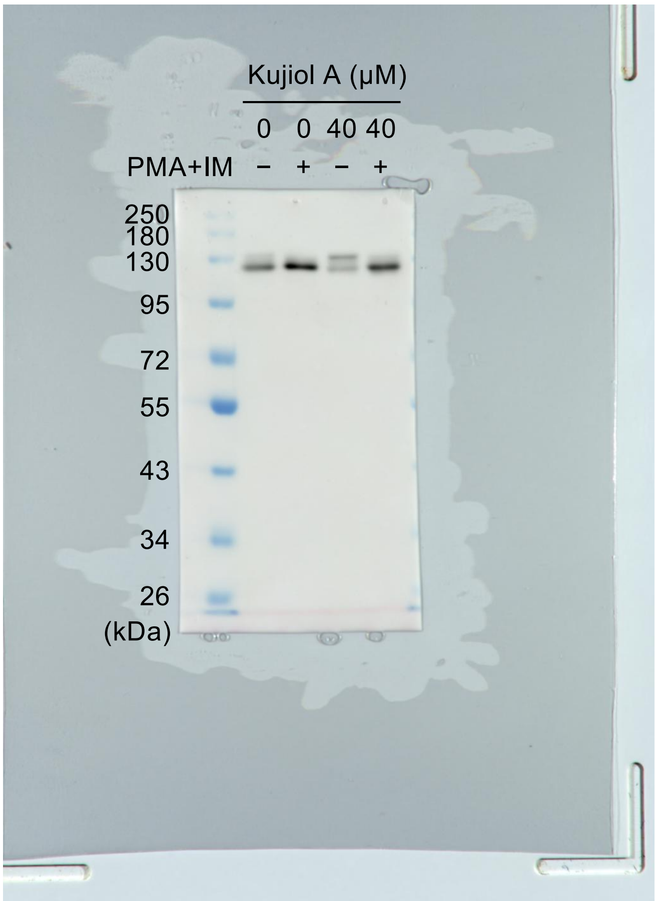

WB: NFATc2

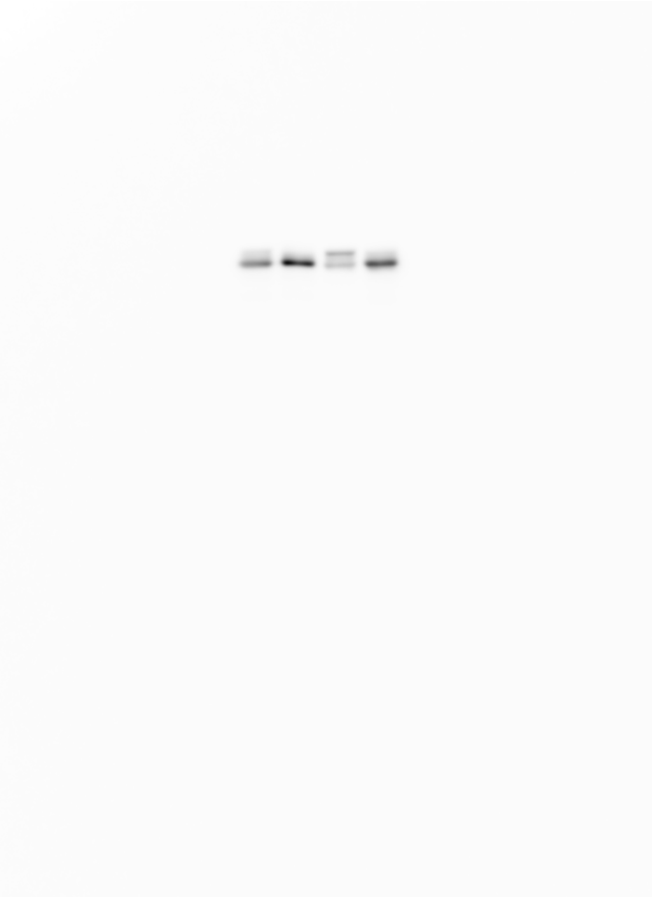

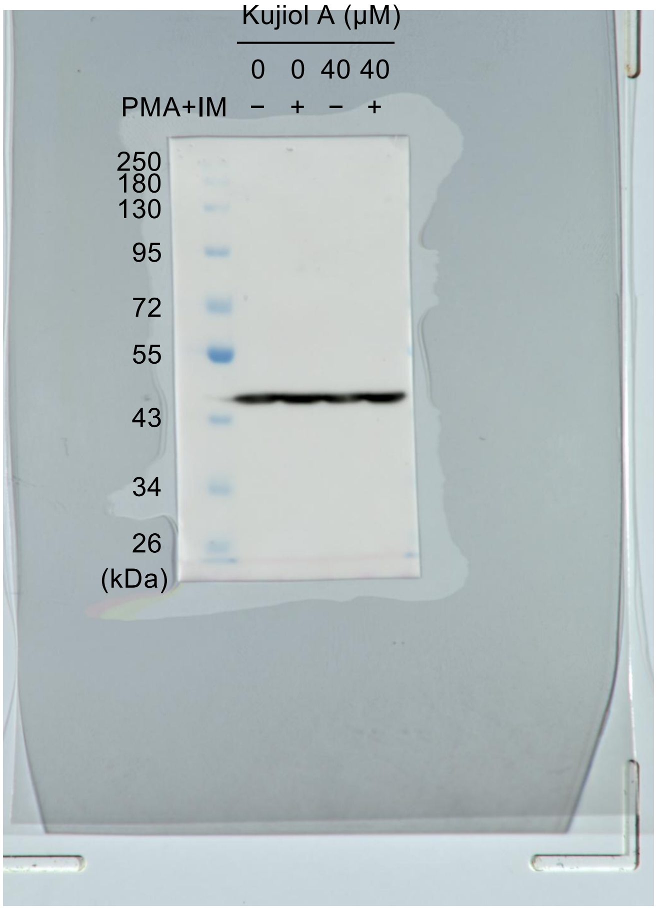

WB: β-Actin (reprobed)

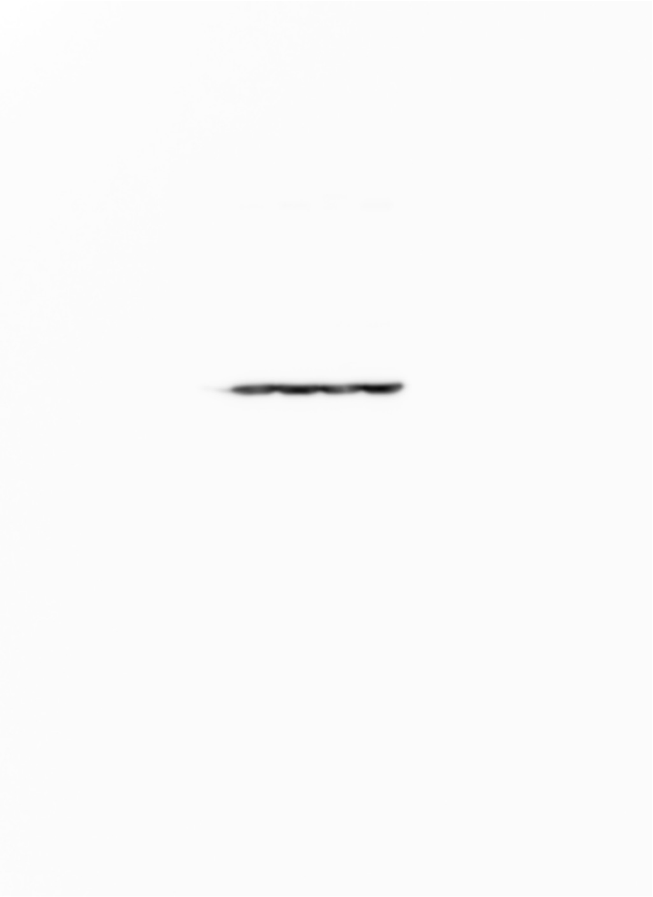

Figure S10: Original blots in Figure 7F (Exp. 1)

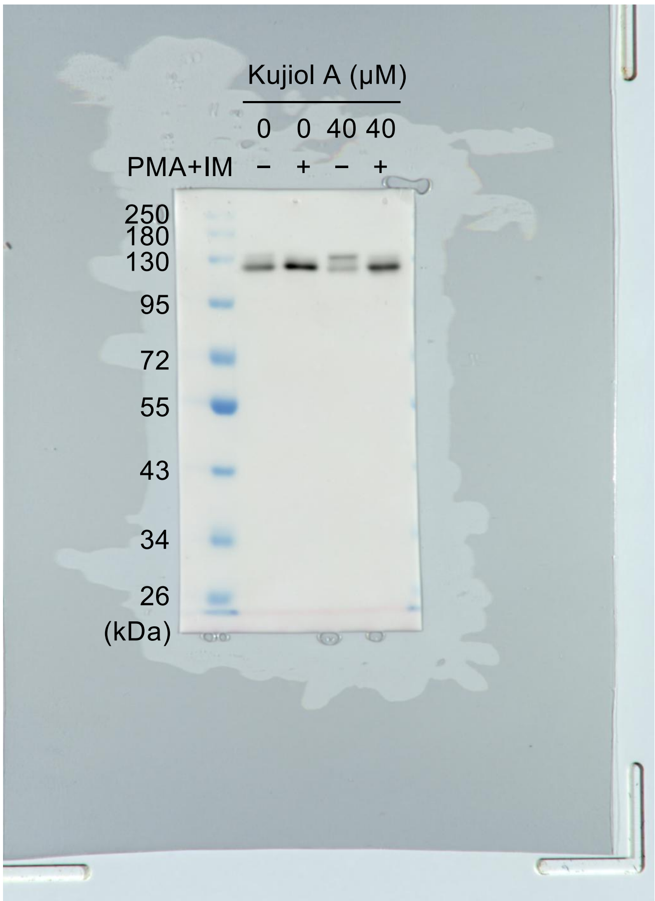

WB: NFATc2

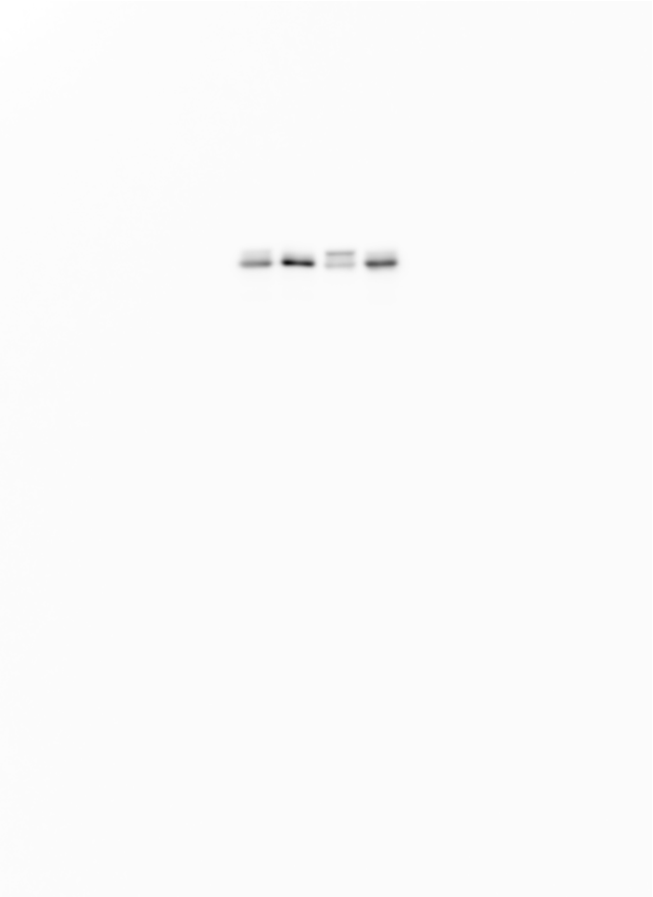

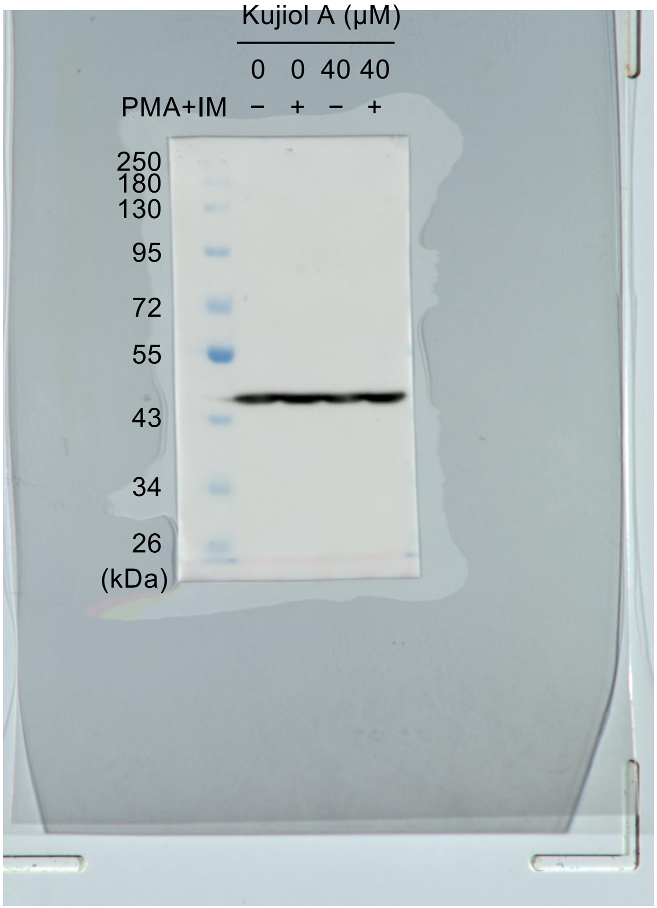

WB: β-Actin (reprobed)

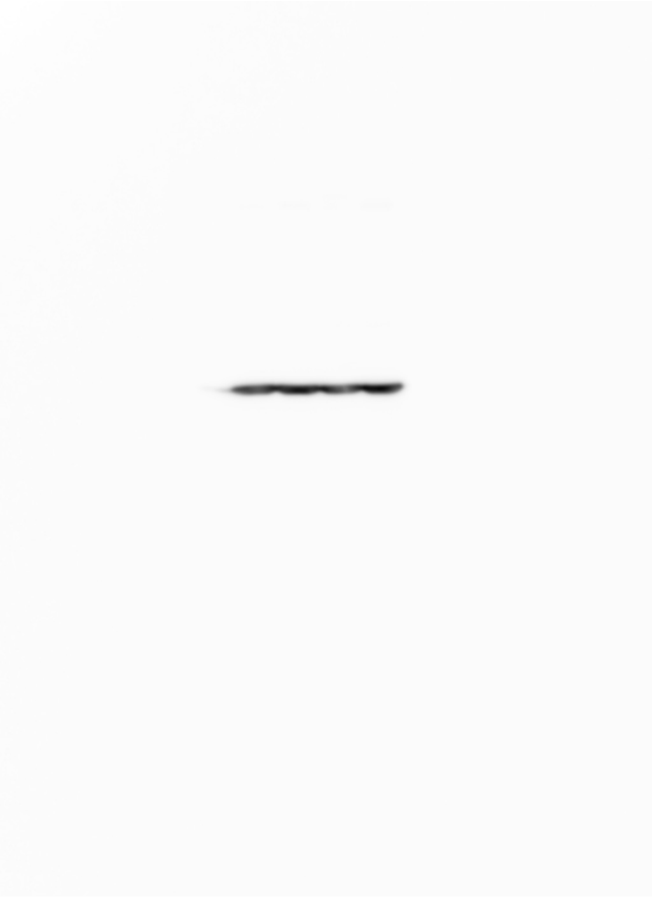

Figure S11: Original blots in Figure 7F (Exp. 2)

WB: NFATc2

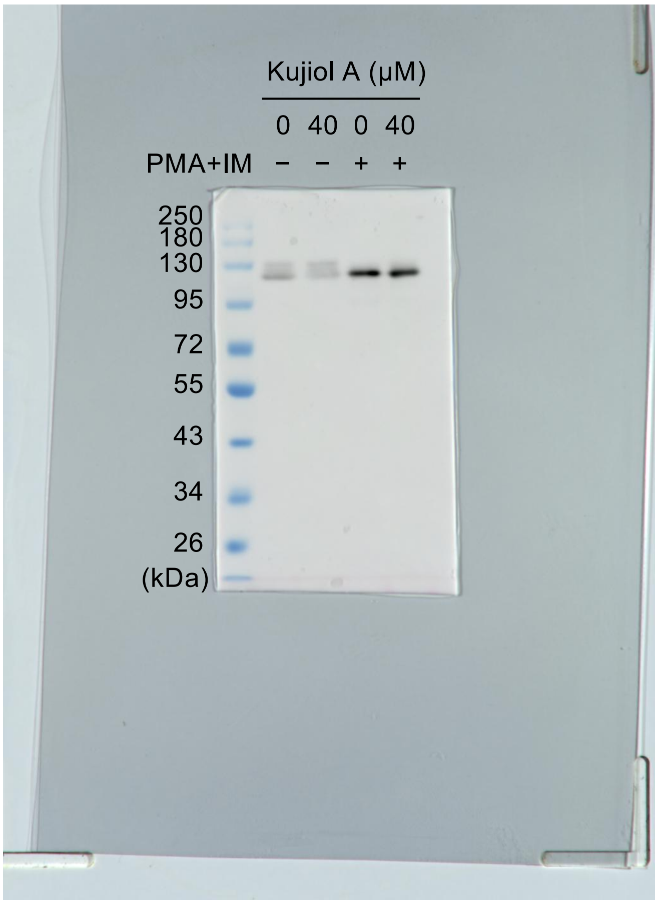

WB: β-Actin (reprobed)

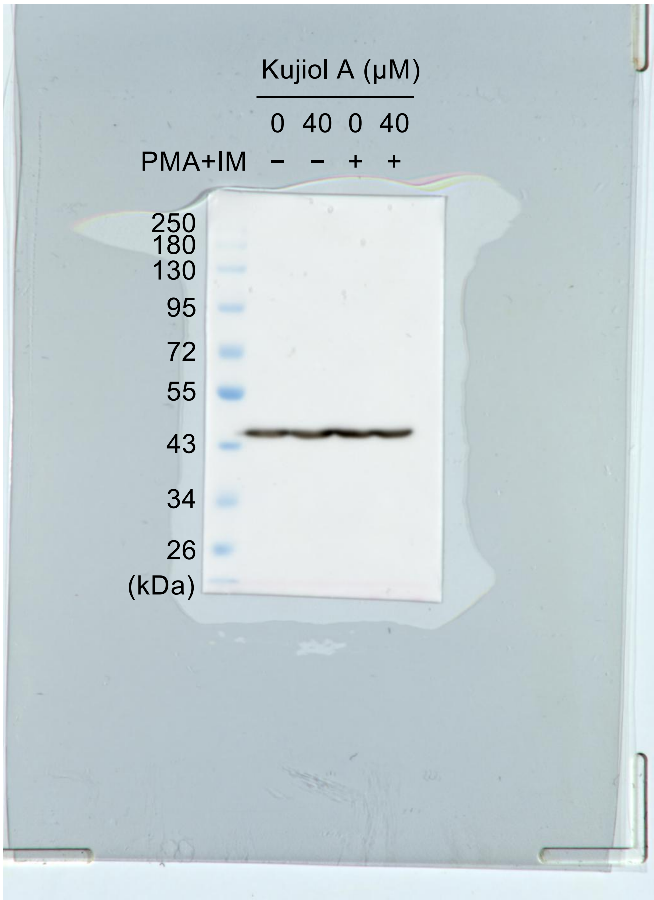

Figure S12: Original blots in Figure 7F (Exp. 3)

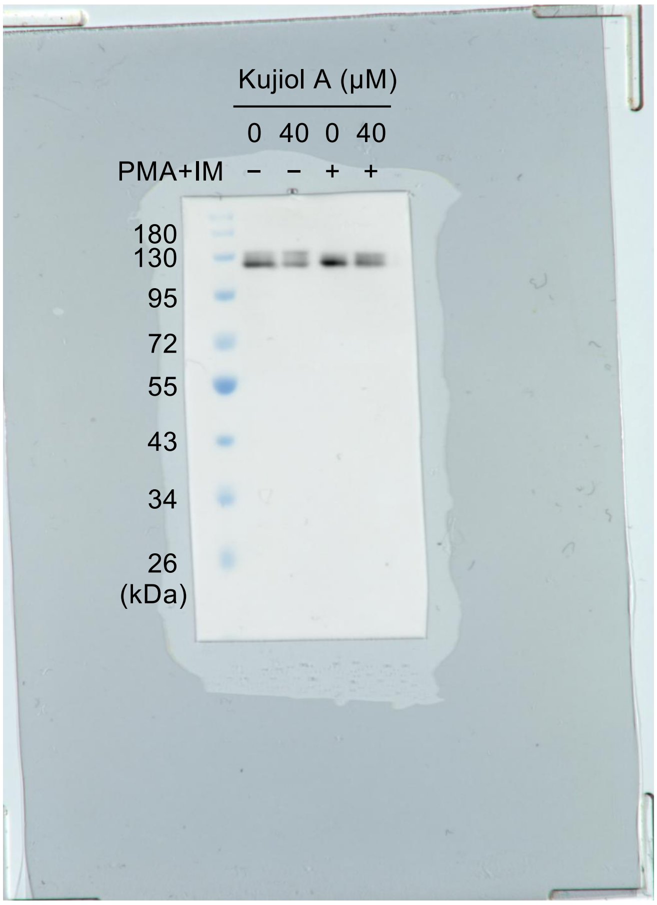

WB: NFATc2

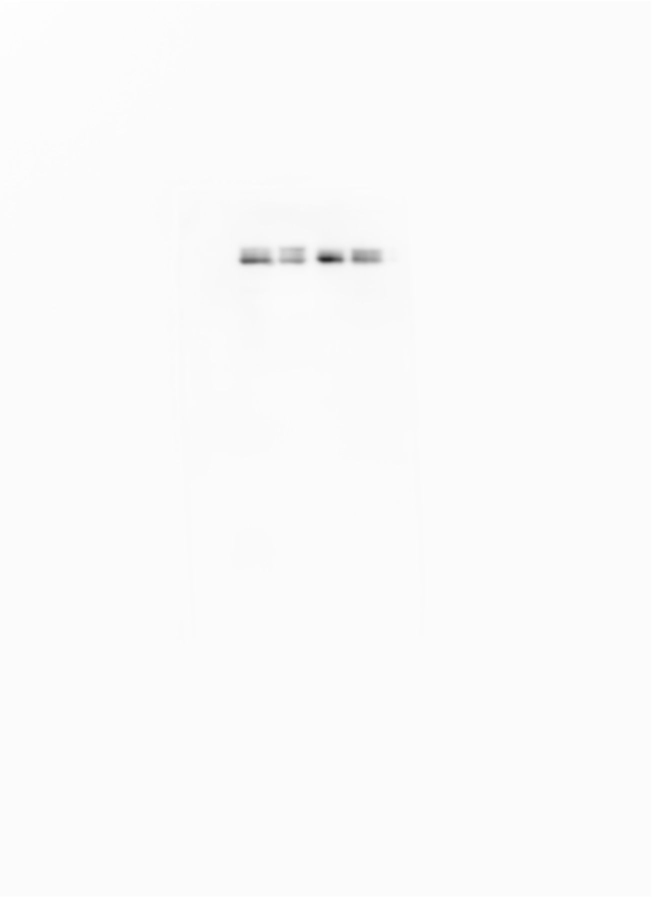

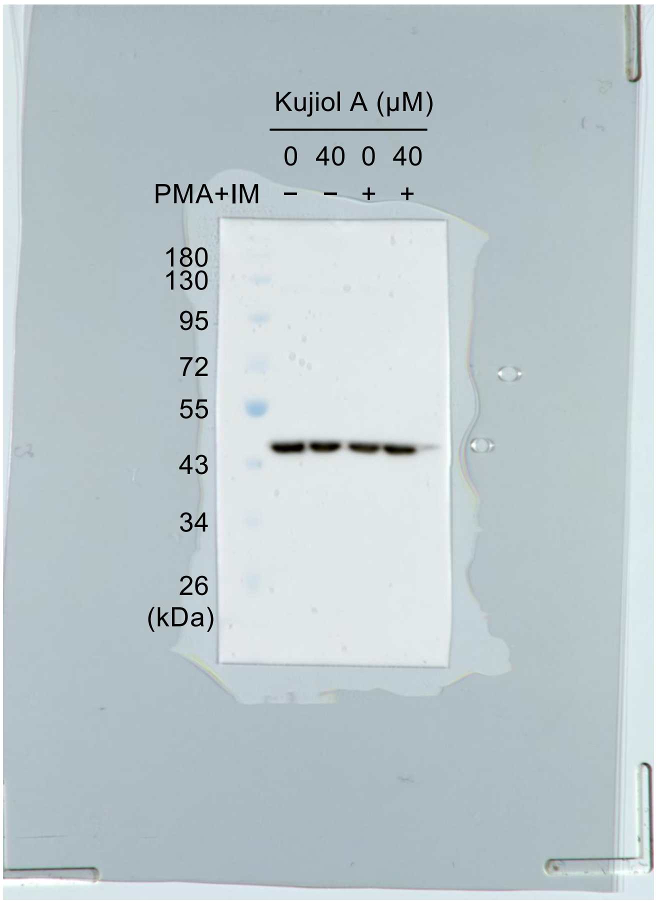

WB: β-Actin (reprobed)

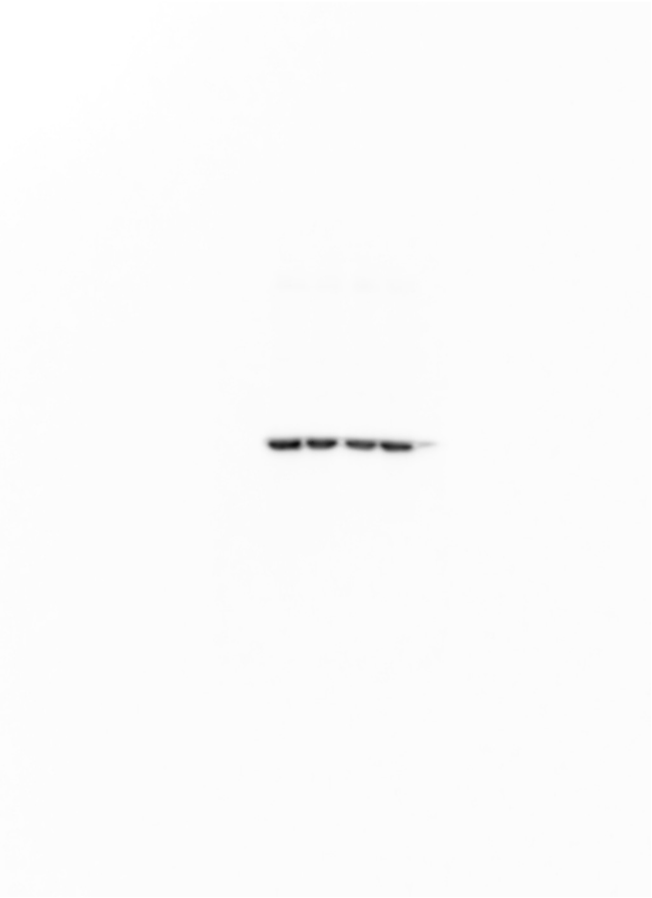

Supplement: Supplementary file 1 [file molecules-31-01613-s001.zip › molecules-4211874-supplementary.pdf]
